# Genomic surveillance of SARS-CoV-2 in US military compounds in Afghanistan reveals multiple introductions and outbreaks of Alpha and Delta variants

**DOI:** 10.1186/s12864-022-08757-5

**Published:** 2022-07-15

**Authors:** Irina Maljkovic Berry, Jun Hang, Christian Fung, Yu Yang, Marcus Chibucos, Adam Pollio, Jay Gandhi, Tao Li, Matthew A. Conte, Grace M. Lidl, Jay A. Johannigman, Heather Friberg

**Affiliations:** 1 Viral Diseases Branch, Walter Reed Army Institute of Research, 503 Robert Grant Ave, Silver Spring, MD 20910 USA; 2 Uniformed Services University of the Health Sciences, Bethesda, MD USA

**Keywords:** SARS-CoV-2, Outbreaks, Afghanistan, Military

## Abstract

**Background:**

With the emergence and spread of SARS-CoV-2 variants, genomic epidemiology and surveillance have proven invaluable tools for variant tracking. Here, we analyzed SARS-CoV-2 samples collected from personnel located at the US/NATO bases across Afghanistan.

**Results:**

Sequencing and phylogenetic analyses revealed at least 16 independent introductions of SARS-CoV-2 into four of these relatively isolated compounds during April and May 2021, including multiple introductions of Alpha and Delta variants. Four of the introductions resulted in sustained spread of the virus within, and in two cases between, the compounds. Three of these outbreaks, one Delta and two Alpha, occurred simultaneously.

**Conclusions:**

Even in rigorously controlled and segregated environments, SARS-CoV-2 introduction and spread may occur frequently.

**Supplementary Information:**

The online version contains supplementary material available at 10.1186/s12864-022-08757-5.

## Background

With 10,637 newly confirmed COVID-19 cases and 382 reported deaths between June 1 and June 7, 2021, Afghanistan was, during this time, experiencing its largest COVID-19 wave since the beginning of the pandemic [1]. In a country of more than 39 million, only 662,003 doses of SARS-CoV-2 vaccines had been administered as of June 15, 2021, mainly due to vaccine inaccessibility and shipping delays [2]. Furthermore, the fragile healthcare and public health systems, due to more than 40 years of prolonged conflict, had been left with limited testing capacities, and as of 15 June, 93,765 SARS-CoV-2 infections had been reported from a total of 534,505 performed tests [3, 4]. However, the Afghanistan Ministry of Health had estimated that close to a third of the country, roughly 10 million people, had by this time contracted the infection [5]. The first case of SARS-CoV-2 was reported in Afghanistan’s Herat Province on February 24, 2020, believed to have been imported from Qom, Iran [6]. Since then, several COVID-19 waves had been observed in the country, with the surge in June suspected to be associated with the increased transmission of the SARS-CoV-2 Delta variant (lineage B.1.617.2) in the region, first seen most notably in India [1].

Afghanistan until recently housed a large population of NATO and US forces, with the Resolute Support Mission to train, advise, and assist the Afghan National Defense and Security Forces (ANDSF) and institutions. In addition to the US and NATO personnel, the joint military compounds in Afghanistan contained a large population of contracted support personnel originating from many different countries such as US, Afghanistan, India, Denmark, Turkey, Uzbekistan, Ukraine, Uganda, Kenya, Pakistan, and others [7]. The compounds, and the personnel within, were generally segregated from the local populace, although prior to the COVID-19 pandemic vetted locals were allowed onto these Forward Operating Bases (FOBs) for the purposes of trade. Since the pandemic, the FOBs largely existed as isolated environments, where travel was restricted to planned transports between FOBs only. Early in the pandemic, personal leave for the military and supporting personnel to their countries of origin was not permitted, however, this restriction was lifted by February 2021.

Deployed US/NATO military environments in Afghanistan consisted of communal living, where Containerized Housing Units and other living spaces, such as showering facilities and dining halls, were commonly shared between two or more individuals. The confined living and working settings, coupled with mixing and travel of individuals from different countries of origin, placed the US Service Members at a greater risk of communicable respiratory infections such as SARS-CoV-2 [7, 8]. Therefore, SARS-CoV-2 mitigation strategies were employed early in the pandemic. These included aggressive quarantining and testing strategies with a minimum 14-day quarantine for arriving personnel at the Hamid Karzai International Airport NATO base, establishment of small quarantine cohorts with regular testing and isolation upon detection of infection, cohort re-quarantine if positive cases were detected during the quarantine time period, and establishment of COVID Task Force to track all arriving individuals, COVID cases and contacts [7]. Furthermore, in order to track SARS-CoV-2 outbreaks and variant spread across the DoD installations, virus sequencing and genomic surveillance were implemented early across the Department of Defense (DoD), and these efforts have provided important insights into the spread of the virus in deployed environments [7, 9]. As part of the DoD’s genomic surveillance efforts, for this study we sequenced and analyzed SARS-CoV-2 samples collected from personnel at major military compounds in Afghanistan.

## Results

### SARS-CoV-2 Alpha and Delta variants of concern were predominant in the Afghanistan specimens

**Fig. 1:**
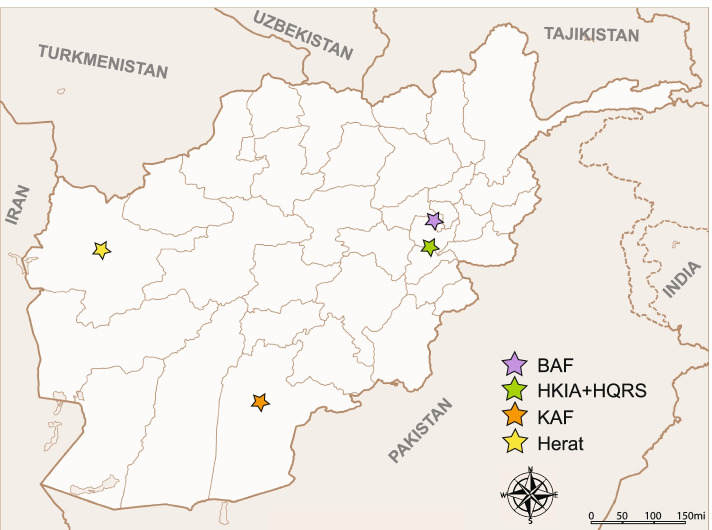
Map of Afghanistan with approximate locations of the sampled FOBs. Map has been adapted from Afghanistan location map.svg [10]

A total of 122 SARS-CoV-2 samples, collected from all positive SARS-CoV-2 cases in four US/NATO military locations in Afghanistan between February 16, 2021 and May 29, 2021, were received for sequencing at the Walter Reed Army Institute of Research. The samples were collected from the compounds at the Hamid Karzai International Airport base and Headquarters in Kabul (HKIA, *n* = 63; HQRS, *n* = 1), Bagram Airfield (BAF, *n* = 31), Kandahar Airfield (KAF, *n* = 25) and Herat (n = 2) (Fig. 1). Of 122 sampled individuals, 15 had been vaccinated prior to their SARS-CoV-2 infection (HKIA = 14, BAF = 1, vaccine information not available), and 5 other exhibited severe disease symptoms (HKIA). There was one instance of a re-infection, one instance of a re-infection subsequent to vaccination, and seven instances of diagnostic test failure (cases with high suspicion of COVID-19 but negative or inconclusive diagnostic results).

**Fig. 2:**
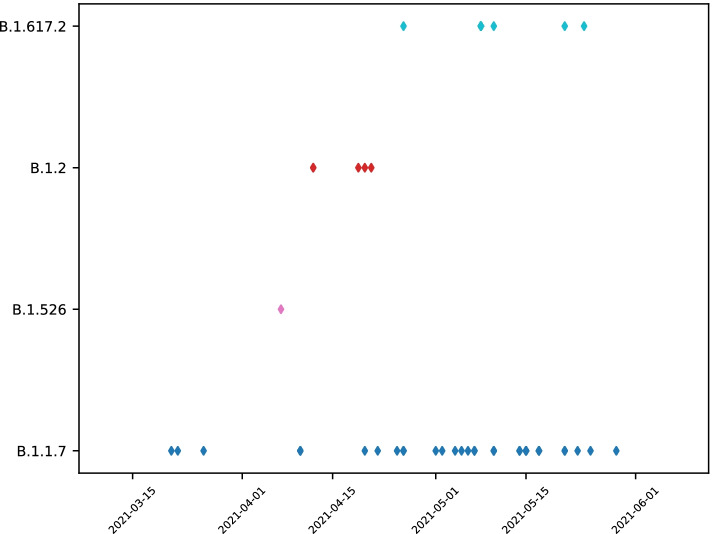
Date and lineage assignment of SARS-CoV-2 genomes with > 80% genome coverage and sample collection date information

Sequencing resulted in 74 complete SARS-CoV-2 genomes and 23 partial genomes (of which 9 had ≥80% genome coverage for downstream analyses). Twenty-five samples did not produce enough data for genome assembly. Of 83 complete and partial genomes with enough information for lineage determination and phylogenic analyses, 76 (91.5%) belonged to the lineages B.1.1.7 (*n* = 56) and B.1.617.2 (*n* = 20). One genome belonged to the lineage B.1.526. The average number of ambiguous positions was low, 0.95 per genome, not expecting to affect the placement of the genomes into their respective lineages. All genome lineages and collection dates are summarized in Table 1 and Fig. 2.

**Table 1:** SARS-CoV-2 genomes and lineage distribution per location

Location	B.1.1.7 (Alpha)	B.1.617.2 (Delta)	B.1.526 (Iota)	B.1.2
HKIA	21	20	0	6
HQRS	1	0	0	0
Herat	2	0	0	0
BAF	20	0	1	0
KAF	12	0	0	0

Only 7 out of 15 vaccine breakthrough cases gave rise to enough virus genetic material and genome assembly information for lineage determination. Six belonged to the B.1.2 lineage, and one belonged to the Delta variant. Two severe disease cases were Delta, and three were Alpha variants. Neither of the reinfection, nor the vaccination + reinfection, cases gave enough genome information for virus lineage determination. Diagnostic test failure viruses belonged to the Alpha (*n* = 3) and Iota (*n* = 1) variants.

### Outbreak and spread of Delta in US/NATO military compounds in Afghanistan

**Fig. 3:**
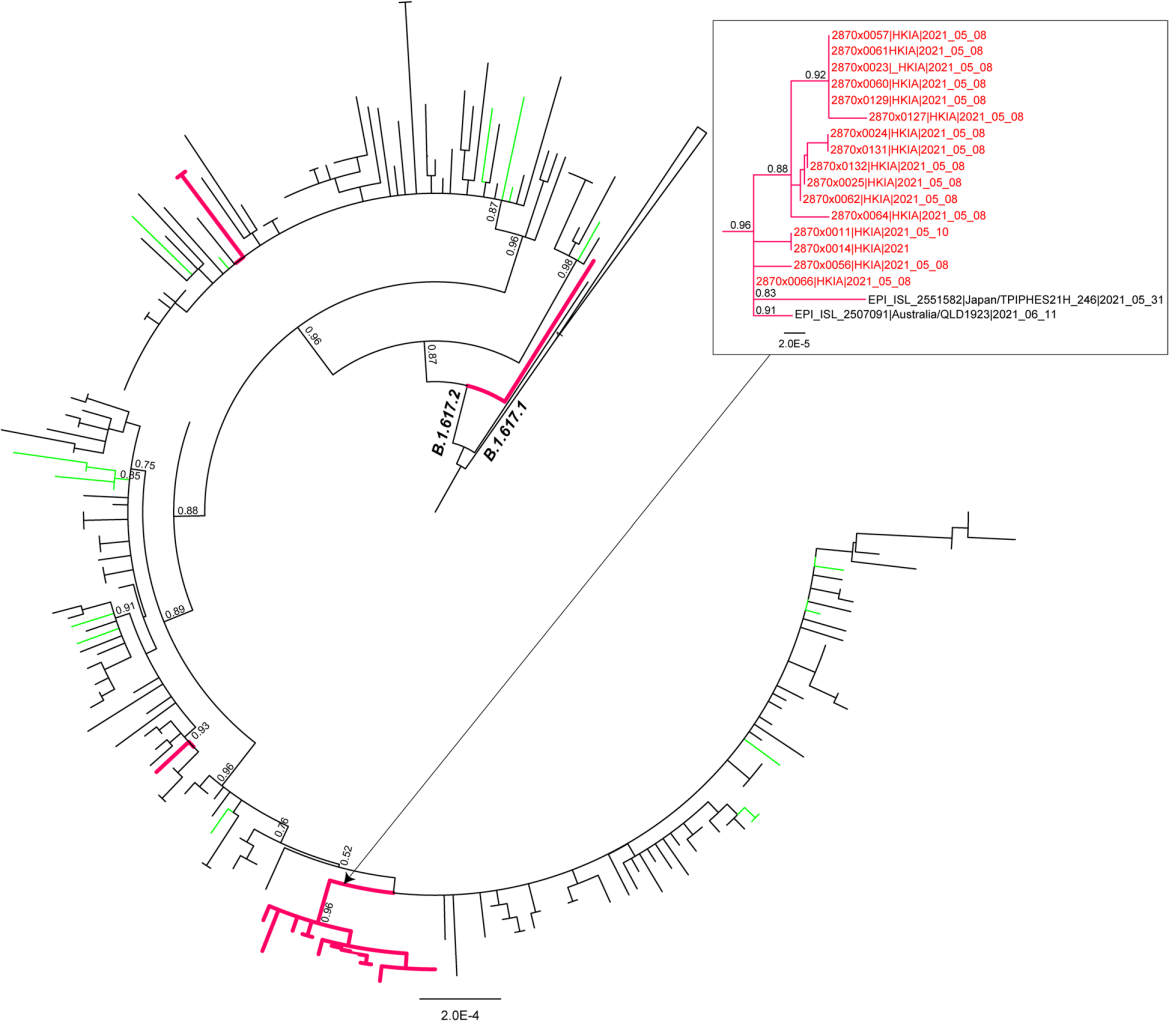
Maximum likelihood tree of the B.1.617 SARS-CoV-2 lineage. Branches leading to genomes obtained from HKIA are marked in red. Probable outbreak at HKIA is magnified. Relevant node confidence values are highlighted. Afghanistan – red; Iran, India, Pakistan – green

Twenty Delta genomes had ≥80% genome coverage and were used in downstream phylogenetic analyses. Sixteen of the genomes, sampled on May 8 and May 10 in HKIA, clustered together in a monophyletic clade in the phylogenetic tree (Fig. 3), indicating their close relatedness and probable outbreak origins within this population. The cluster also contained two reference genomes, one from Japan and one from Australia. Two of our Afghanistan genomes collected on May 21 and May 24 clustered together, separate from the large monophyletic cluster, indicating their independent origins from the main cluster and their close relatedness to each other. Two additional genomes, collected on April 26 and May 8, clustered independently in the tree. These results indicate at least four separate introductions of Delta into the HKAI location, of which at least one appears to have resulted in sustained local transmission. Given the history of SARS-CoV-2 introduction from Iran into Afghanistan [6], Delta genomes from neighboring countries of Afghanistan and India were highlighted in the tree. None of our Delta variant genomes isolated from individuals in Afghanistan clustered closely together with the Delta genomes from the neighboring countries of Iran, Pakistan or India.

### Outbreaks and spread of alpha in US/NATO military compounds in Afghanistan

**Fig. 4:**
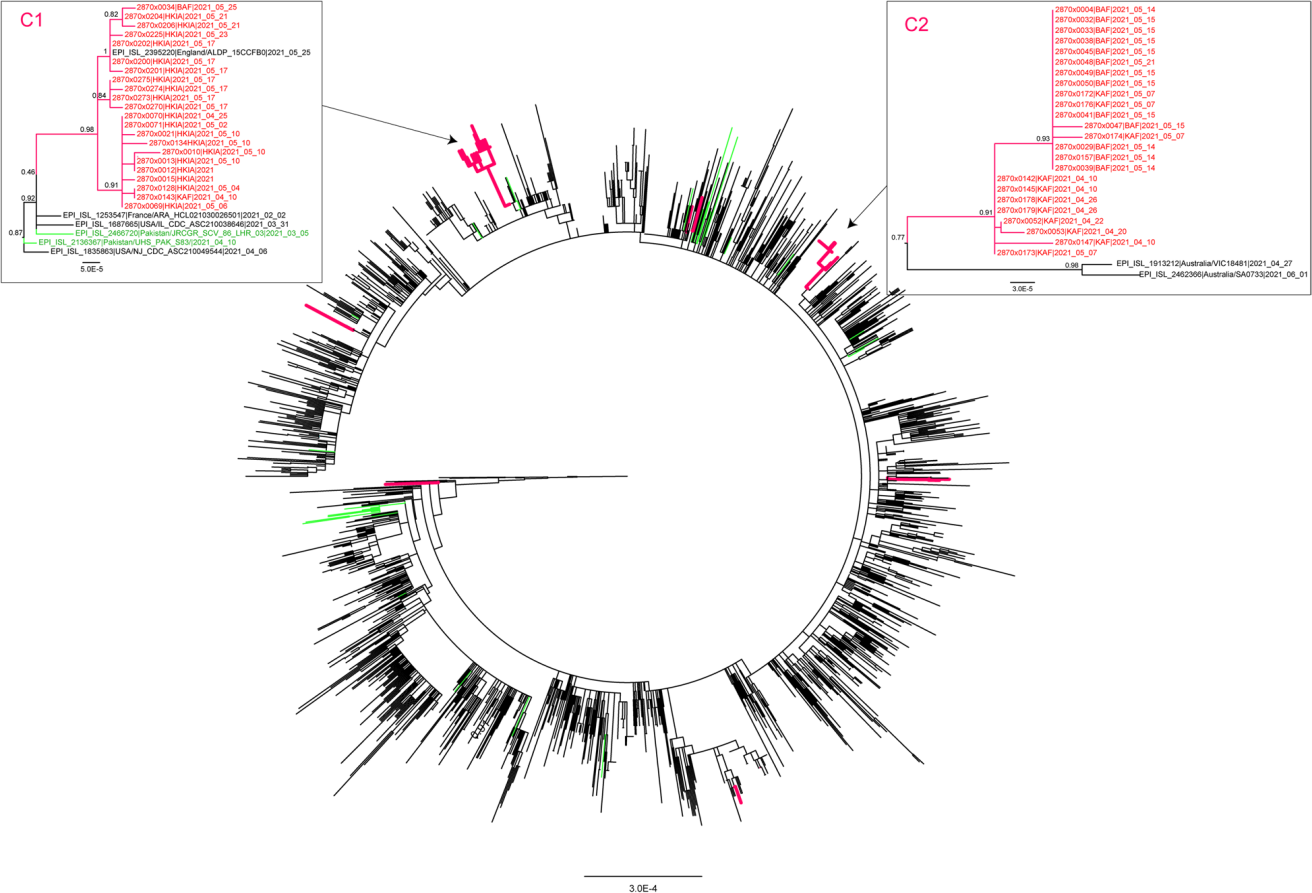
Maximum likelihood tree of the B.1.1.7 SARS-CoV-2 lineage. Branches leading to genomes collected from HKIA, HQRS, Herat, BAF and KAF are marked in red. Relevant node confidence values are highlighted. Afghanistan – red; Iran, India, Pakistan – green. Probable outbreaks are magnified

Fifty-six Alpha genomes had ≥80% genome coverage and were used in downstream phylogenetic analyses. Presence of N501Y S mutation was confirmed in all the complete Alpha genomes except in one genome collected at BAF on May 15, 2021. Instead, this genome had the L452R mutation in its Spike protein. Because L452R is characteristic of the Delta variant, and because both Alpha and Delta were circulating in Afghanistan at the same time, we investigated whether this genome was a possible recombinant of the two variants. Our RDP4 results indicated no presence of recombination between the two lineages in this genome. A phylogenetic tree of Alpha revealed two large monophyletic clusters containing the majority of our sequenced Alpha genomes (Fig. 4). One cluster contained 22 genomes (C1, Fig. 4), of which 20 were from HKAI collected between April 25 and May 23. One genome was from KAF collected on April 10, and one genome was from BAF collected on May 25. Based on sample collection data, the single genome from KAF predated all the HKIA genomes, suggesting possible transmission from KAF to HKIA leading to the Alpha variant outbreak at HKIA, and spillover into BAF. The only background reference genome in this cluster was from England, collected on May 25. Contracting personnel from the UK was present within the HKIA population.

A second monophyletic cluster contained 24 of our sequenced genomes (C2, Fig. 4), 13 of which were collected at BAF May 14–21, and 11 collected at KAF between April 10 and May 7. This indicated that genomes from this outbreak at KAF and BAF are related, and accounting for sampling times, that KAF might have seeded the introduction of Alpha into BAF, which was followed by sustained transmission in this location. Additionally, 2 small clusters of 2 samples each, BAF + BAF and BAF + HQRS, were also observed in the tree, with the remaining samples individually separated throughout the B.1.1.7 lineage. One of these genomes located basally to the B.1.1.7 lineage. This was the same genome that contained L452R but not the typical Alpha N501Y mutation in its Spike protein. Our results indicate at least 10 independent introductions of Alpha into these Afghanistan locations during the surveillance period. None of our Alpha genomes clustered closely together with the Alpha genomes from the neighboring countries of Iran, Pakistan or India.

### Other SARS-CoV-2 lineages in US military compounds in Afghanistan

Seven remaining genomes with ≥80% genome coverage fell within the B.1.526 lineage (*n* = 1, BAF, April 2, 2021), and the B.1.2 lineage (*n* = 6, HKIA, 12–21 April, 2021). Afghanistan genomes from the B.1.2 lineage clustered together in a monophyletic cluster suggesting sustained transmission of this virus in the HKIA population during this time (Additional file 1).

## Discussion

The genetic variability of SARS-CoV-2 has been closely followed since the beginning of the pandemic, with a major concern of the virus obtaining mutations that may increase its virulence, transmissibility, and escape from immune responses. These fears were realized in December 2020, with the emergence and rapid global spread of a variant characterized by an unusually large number of nucleotide substitutions, [11, 12]. The variant, named Alpha (B.1.1.7 lineage), has been associated with an increase in transmissibility and disease severity [13, 14]. Alpha was closely followed by the emergence of other SARS-CoV-2 variants, some of which exhibit additional phenotypic changes of concern, such as escape from neutralization by antibodies derived from convalescent sera, vaccinated individuals, or antibodies used in COVID-19 therapy [15–18]. Therefore, careful monitoring of SARS-CoV-2 variant spread is of utmost importance, as it can inform of the need for adjustment of prevention and control strategies and provide further insight into potential vaccine escape.

Delta variant (B.1.617.2 lineage) first emerged in late 2020, exhibiting both increased transmissibility and potential to reduced neutralization by SARS-CoV-2 specific antibodies, and has been associated with modest reduction in vaccine efficacy [17–20]. Following its explosive spread in India, Delta rapidly dispersed throughout the world, which prompted adjusted public health responses such as a delay in lifting movement restrictions in the United Kingdom, and increased travel restrictions in many countries in Europe. In Afghanistan, the June 2021 surge in COVID-19 cases prompted reinstatement of local lockdown measures, such as school and workplace closures. Even though the SARS-CoV-2 spread in Afghanistan at that time had not been tied to the Delta variant, its presence had been reported in Pakistan and Iran, from which cross—border traffic with Afghanistan is common, and which previously resulted in SARS-CoV-2 transmission [6, 21]. Furthermore, Afghanistan’s increased geopolitical ties with its regional neighbor India, augmented by the departure of the US troops from this region, further increased the concerns of Delta being the cause of this COVID-19 wave.

We collected and sequenced SARS-CoV-2 genomes from the military and support personnel from four different locations in Afghanistan, and in these unique populations with tight restrictions and high level of health protection measures, we show multiple introductions and spread of both Alpha and Delta variants, as well as presence of Iota. This is the largest set of SARS-CoV-2 genomes from Afghanistan, and the earliest genomes of the Delta variant from the country. The genomic surveillance was performed on samples collected between April and May 2021, and during this time, one outbreak of the Delta variant, two separate outbreaks of the Alpha variant, and one outbreak of lineage B.1.2 were observed, as well as 12 additional introductions of SARS-CoV-2.

The Delta variant outbreak occurred in May and was contained within one military location only, HKIA. The only two reference genomes within the Delta Afghanistan cluster, from Japan and Australia, were collected subsequent to our genomes, suggesting transmission of this virus outside of Afghanistan as well. In addition to the Delta outbreak, a simultaneous outbreak of the Alpha variant was observed at HKIA. However, the Alpha variant outbreak was not contained within the HKIA location only, suggesting possible transmission from KAF to HKIA, leading to the Alpha variant outbreak at HKIA, as well as spillover to BAF. The reference genome from England within this Alpha clade was collected subsequently to the HKIA samples, suggesting additional transmission of this variant outside of Afghanistan. Altogether, there were at least 7 SARS-CoV-2 introductions into the HKIA location, resulting in three outbreaks (Delta, Alpha and B.1.2), during the studied period of time. An additional outbreak of the Alpha variant, independent from the Alpha at HKIA, was observed at KAF and BAF locations. The outbreak started at KAF in April, followed by dissemination to and sustained spread at BAF in May. In total, there were at least 8 independent introductions of SARS-CoV-2 into BAF, with one sustained spread of the Alpha variant, and at least two independent introductions of SARS-CoV-2 into KAF, with one Alpha outbreak at the location. Finally, the two genomes from Herat clustered independently in the tree, suggesting at least two separate introductions of Alpha variant to the Herat FOB.

Even though all positive SARS-CoV-2 cases from the US/NATO compounds in Afghanistan were obtained, not all samples were successfully sequenced, which lowered the number of available genomes and limited resolution of this study. However, the majority of failed samples came from KAF and were collected during the Alpha outbreak at KAF and BAF, making it more likely they too were part of the already reported Alpha variant outbreak within these locations.

Another study limitation was the lack of SARS-CoV-2 genomes from the local Afghanistan population during this period, making it impossible to determine whether some of the introductions into the US/NATO military compounds were related to locally circulating SARS-CoV-2 strains. Given the segregated nature of these compounds it is possible no connection exists; however, the observed circulation of SARS-CoV-2 variants further highlights the importance of expanded genomic surveillance in this region. We did not observe close genetic clustering of our genomes to genomes from Afghanistan’s neighboring countries or India. Nevertheless, our results point to transmission of the virus between the FOBs leading to subsequent compound outbreaks. Travel between FOBs was restricted but not uncommon, and often included mission-related travel as well as leave-related travel. The communal nature of living environments in these locations, both for temporary transit and for more permanent housing purposes, aided in virus dispersal between the FOBs, and in sustained transmissions within the FOBs. For instance, despite rigorous testing and quarantine strategies, cases of SARS-CoV-2 infection had been identified in individuals recently released from quarantine at HKIA that had subsequently travelled to other FOBs [7]. Thus, improved prevention and control strategies in compounds of this nature, such as smaller quarantine cohorts or individual quarantine quarters, and increased social distancing and hand hygiene, may be important to consider. Defining sustained local transmission as 3 or more monophyletically connected genomes, we notice that the majority of our detected virus introductions (12 out of 16) did not lead to sustained outbreaks in these locations. Even though it is possible that asymptomatic undetected cases existed, many asymptomatic cases were identified due to the implemented COVID tracking, quarantine, and testing policies [7]. These results would indicate that the prevention and control strategies, albeit not completely successful, aided in preventing the spread of SARS-CoV-2 in these environments.

Recombination is common in coronaviruses, and has also been reported for SARS-CoV-2 [22, 23]. Although our analyses did not yield a recombination signal between Alpha and Delta in a single genome containing mutations from both variants, a micro-recombination event cannot be excluded. Nevertheless, this mutation pattern was not detected in any other samples. This may be due to this mutation combination not resulting in increased fitness for onward transmission and/or that this individual isolated and did not transmit to others.

## Conclusions

Our results show frequent introductions of Alpha and Delta variants into the US/NATO military compounds during a period of 2 months in Afghanistan, as well as transmission of viruses between the compounds. Only a fraction of introductions resulted in sustained local spread of both Alpha and Delta variants of SARS-CoV-2 in these populations. This combination of genomic and epidemiologic surveillance provides insights into the local dynamics of SARS-CoV-2 spread and provides actionable results that can be used for adjustment of local prevention and control strategies.

## Methods

### Sample collection

All samples were collected under a Public Health determination protocol # 2870, as reviewed by the Walter Reed Army Institute of Research (WRAIR) Human Subject Protection Branch, and all experiments were performed in accordance with relevant guidelines and regulations. The nasopharyngeal (NP) respiratory specimens were collected using Ultra-thin minitip flocked swabs (Catalog number 560C; COPAN Diagnostics Inc., Murrieta, CA). A positive PCR test for SARS-CoV-2 was re-swabbed the next day to 1) repeat the PCR and 2) obtain a fresh swab to be placed into the PrimeStore Molecular Transport Medium (MTM) (Longhorn Vaccines and Diagnostics LLC, San Antonio, TX). The PrimeStore MTM media was used due to the non-availability of ultra-cold storage chain at dispersed locations within the deployed locations. The samples were stored in refrigerators prior to shipping, at ambient temperatures from Afghanistan to the US, and then stored at -80 °C at the WRAIR. Breakthrough infections were defined as infections that occurred more than 2 weeks post vaccination. Re-infection cases were defined as greater than 90 days from a previous case onset date or first positive specimen.

### Sequencing and genome assembly

The nucleic acids extraction from the NP specimens in MTM was conducted using the KingFisher Flex System and MagMAX Pathogen RNA/DNA Kit (Thermo Fisher Scientific, Pittsburgh, PA) or the QIAamp Viral RNA Extraction Kit by following the manual or Qiacube protocols from the manufacturer (QIAGEN, Germantown, MD). The purified RNA samples were subjected to one-step RT-PCR amplification of SARS-CoV-2 genome using the Fluidigm Juno nucleic acids amplification system (Fluidigm Corporation, South San Francisco, CA) followed by NGS library preparation using Illumina DNA Prep Kit (Illumina, San Diego, CA). Alternatively, the QIAseq DORECT SARS-CoV-2 Kit was used for whole genome RT-PCR amplification and NGS library preparation. The NGS libraries were sequenced using the MiSeq System and MiSeq Reagent Kit v3 (600-cycle) (Illumina). Due to the importance of each specimen, we processed and attempted sequencing of every sample regardless of its CT value [24]. Reads from each of the Afghanistan Sars-CoV-2 samples were mapped to the Wuhan-Hu1 reference (NCBI accession number NC_045512.2). For the mapping and analysis, we used a modified version of NGS_Mapper with Pilon for more accurate indel determination [25, 26]. The default setting of 20/80 was used for NGS_Mapper variant calling, allowing for analyses of variant frequencies per nucleotide position down to 20%. The minimum nucleotide Phred score threshold was set to 25 and the minimum depth of coverage to detect variants was set to 10x. The consensus genomes were quality checked and manually curated using IGV and Geneious version R10, as well as each sample’s VCF file for statistical support [27, 28]. This post-assembly cleaning process of the final consensus also ensured removal of any nucleotide variants present due to primer-induced error (variants only occurring in the known primer regions and only found at the ends of the reads).

### Phylogenetic and recombination analyses

Sequences with 80% or more genome coverage were used in downstream phylogenetic analyses. Of these, sequences with 15 or more mixed (ambiguous) non-N positions were removed from phylogenetic analyses, as this may be indicative of contamination or possible dual infections. Using a script-based pipeline to automate analyses, our genome sequences in FASTA format were queried against a BLAST database generated from SARS-CoV-2 sequences downloaded via the GISAID API on 2021-06-03. The top 30 matches with e-value less than 1e-250 and percent identity greater than 99.9% were retained for each query sequence. All GISAID sequences originating from Afghanistan were added to the top-BLAST genomes and our sequenced genomes, as were reference genomes from the NextStrain Global set [29]. Duplicate reference genomes and those containing more than 40% non-AGCT characters (including dashes and Ns) when aligned relative to the reference were removed, for a final alignment of 4595 genomes (Additional file 2). All genomes were aligned against the reference NC_045512.2 using MAFFT, manually inspected using Geneious or Jalview, and sites were masked in the alignment according to De Maio et al [28, 30–32]. A maximum likelihood phylogenetic tree was built using FastTree (compiled with -DUSE_DOUBLE option for accurate branch lengths) with the -gtr and -gamma options enabled, as determined by ModelFinder [33, 34]. Node confidence values were determined using the SH-aLRT approach. Clades with node support values ≥0.75 were considered as clusters with adequate support. Local outbreak clusters were defined as well supported monophyletic or nearly monophyletic clades (> 85% of genomes from our study). Recombination testing was performed using RDP4, employing the GENECONV, Bootscan, Maxchi, Chimaera, SiSscan, PhylPro, LARD and 3Seq recombination screening methods on an alignment of the query genomes and reference genomes from Alpha (*n* = 244), Delta (*n* = 68) and other background lineages (*n* = 27) (Additional file 3) [35]. Recombination signal was considered present with significant signal found by three or more employed methods.

## Supplementary Information

**Additional file 1.**

**Additional file 2.**

**Additional file 3.**

## Data Availability

The datasets generated and/or analyzed during the current study are available in the GISAID database under accession numbers EPI_ISL_4572739 - EPI_ISL_4572808, EPI_ISL_4583368, EPI_ISL_4583378, EPI_ISL_4583383, EPI_ISL_4583390, EPI_ISL_4583400, EPI_ISL_4583409, EPI_ISL_4583417, EPI_ISL_4583424, EPI_ISL_4583429, EPI_ISL_4583436, EPI_ISL_4583444, EPI_ISL_4583455 and EPI_ISL_4583464.

## Abbreviations

ANDSF: Afghan National Defense and Security Forces
BAF: Bagram Airfield
BLAST: Basic Local Alignment Search
COVID-19: Coronavirus Disease of 2019
DNA: Deoxyribonucleic Acid
FOB: Forward Operating Base
GISAID: Global Initiative on Sharing Avian Influenza Data
HKIA: Hamid Karzai International Airport
HQRS: Headquarters
KAF: Kandahar Airfield
MTM: Molecular Transport Medium
NATO: North Atlantic Treaty Organization
NCBI: National Center for Biotechnology Information
NGS: Next generation Sequencing
NP: Nasopharyngeal
PCR: Polymerase Chain Reaction
RNA: Ribonucleic Acid
RT-PCR: Real Time Polymerase Chain Reaction
SARS-CoV-2: Severe Acute Respiratory Syndrome Coronavirus 2
US: United States
WRAIR: Walter Reed Army Institute of Research
