# Supplementary figures and images for: Genomic surveillance of SARS-CoV-2 in US military compounds in Afghanistan reveals multiple introductions and outbreaks of Alpha and Delta variants
Source: BMC Genomics. 2022 Jul 15;23:513. doi: 10.1186/s12864-022-08757-5 (PMC9288047; doi:10.1186/s12864-022-08757-5)

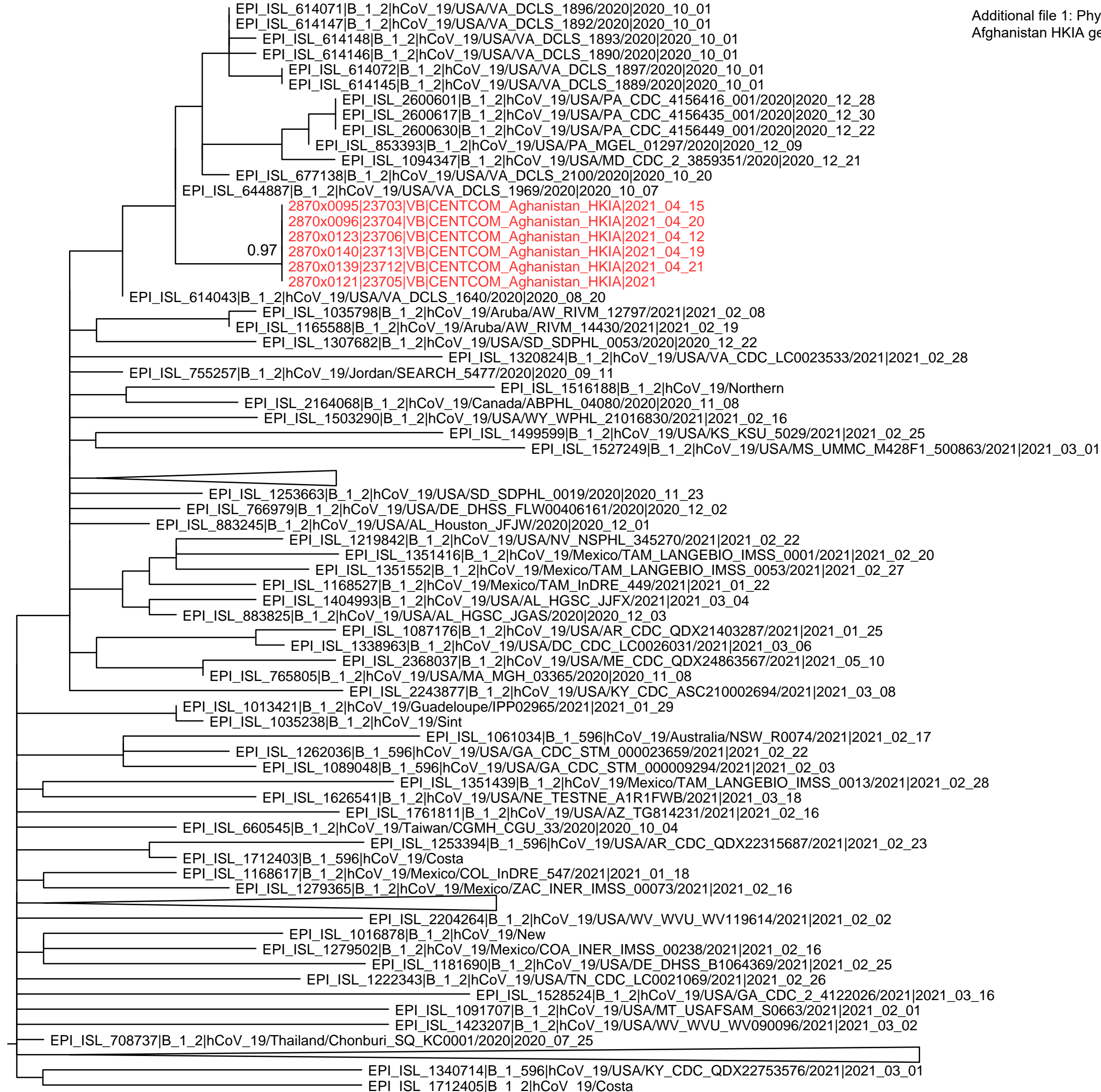

Supplement: Supplementary file 1 — Additional file 1. [file 12864_2022_8757_MOESM1_ESM.pdf]
