# Supplementary material for: Genomic surveillance of SARS-CoV-2 in US military compounds in Afghanistan reveals multiple introductions and outbreaks of Alpha and Delta variants
Source: BMC Genomics. 2022 Jul 15;23:513. doi: 10.1186/s12864-022-08757-5 (PMC9288047; doi:10.1186/s12864-022-08757-5)
Supplement: Supplementary file 3 — Additional file 3. [file 12864_2022_8757_MOESM3_ESM.pdf]

We gratefully acknowledge the following Authors from the Originating laboratories responsible for obtaining the specimens, as well as the Submitting laboratories where the genome data were generated and shared via GISAID, on which this research is based.

All Submitters of data may be contacted directly via www.gisaid.org

Authors are sorted alphabetically.

Accession ID	Originating Laboratory	Submitting Laboratory	Authors
EPI_ISL_1000998, EPI_ISL_1000999, EPI_ISL_1001000, EPI_ISL_1001001, EPI_ISL_1001002, EPI_ISL_1001003, EPI_ISL_1001004	Bundeswehr Institute of Microbiology	Bundeswehr Institute of Microbiology	Markus Antwerpen, Alexandra Rehn, Mathias Walter, Malena Bestehorn-Willmann, Sabine Zange, Enrico Georgi, Roman Wölfel
EPI_ISL_1034755, EPI_ISL_1034756, EPI_ISL_1034757, EPI_ISL_1034758, EPI_ISL_1034759	Bundeswehr Institute of Microbiology	Bundeswehr Institute of Microbiology	Markus Antwerpen, Alexandra Rehn, Mathias Walter, Malena Bestehorn-Willmann, Mike Pillukat, Sabine Zange, Enrico Georgi, Roman Wölfel
EPI_ISL_1034760	Bundeswehr Institute of Microbiology	Bundeswehr Institute of Microbiology	Markus Antwerpen, Alexandra Rehn, Mathias Walter, Malena Bestehorn-Willmann, Sabine Zange, Enrico Georgi, Roman Wölfel
EPI_ISL_1040214	University Hospitals of Geneva, Laboratory of Virology	HUG, Laboratory of Virology and the Health2030 Genome Center	Samuel Cordey, Ana Rita Goncalves, Laurent Kaiser, Lorenzo Cerutti, Henri Pegeot, Melyssa Elies, Deborah Penet, Keith Harshman, Ioannis Xenarios, Emmanouil Dermitzakis
EPI_ISL_1046791, EPI_ISL_1046792, EPI_ISL_1046793	Bundeswehr Institute of Microbiology	Bundeswehr Institute of Microbiology	Markus Antwerpen, Alexandra Rehn, Mathias Walter, Malena Bestehorn-Willmann, Mike Pillukat, Sabine Zange, Enrico Georgi, Roman Wölfel
EPI_ISL_1102188	Lighthouse Lab in Glasgow	Wellcome Sanger Institute for the COVID-19 Genomics UK (COG-UK) Consortium	Harper VanSteenhouse, Yumi Kasai, David Gray, Carol Clugston, Anna Dominiczak and Alex Alderton, Roberto Amato, Jeffrey Barrett, Sonia Goncalves, Ewan Harrison, David K. Jackson, Ian Johnston, Dominic Kwiatkowski, Cordelia Langford, John Sillitoe on behalf of the Wellcome Sanger Institute COVID-19 Surveillance Team
EPI_ISL_1184124	Kentucky State Public Health Lab	Kentucky State Public Health Lab	Stephanie Lunn, Karim George, Joshua Tobias, William Grooms, Vaneet Arora, Matthew Johnson, Rachel Zinner, Rhonda Lucas
EPI_ISL_1191831	National Microbiology Reference Laboratory	Quadram Institute Bioscience	Tapfumanei Mashe, Faustinos T Takawira, Hlanai Gumbo, Kenneth K Maeka, Agnes Juru, Charles Nyagupe, Sekesai Zinyowera, Muchaneta Mugabe, Thanh Le Viet, Justin O'Grady, Gemma Kay, David Baker, Gaetan Thilliez, Ana-Victoria Gutierrez, Robert Kingsley, Leonardo de Oliveira Martins, Andrew Tarupiwa, Andrew J. Page, Raiva Simbi
EPI_ISL_1257407, EPI_ISL_1293930, EPI_ISL_1294567, EPI_ISL_1294589, EPI_ISL_1294690, EPI_ISL_1294810, EPI_ISL_1295034	Lighthouse Lab in Glasgow	Wellcome Sanger Institute for the COVID-19 Genomics UK (COG-UK) Consortium	Harper VanSteenhouse, Yumi Kasai, David Gray, Carol Clugston, Anna Dominiczak and Alex Alderton, Roberto Amato, Jeffrey Barrett, Sonia Goncalves, Ewan Harrison, David K. Jackson, Ian Johnston, Dominic Kwiatkowski, Cordelia Langford, John Sillitoe on behalf of the Wellcome Sanger Institute COVID-19 Surveillance Team
EPI_ISL_1300479	Genetica Molecular and Subdepartamento de Virologia ISP Chile	Instituto de Salud Publica de Chile	Javier Tognarelli, Karen Orostica, Barbara Parra, Loredana Arata, Jaime Lagos, Gisselle Barra, Patricia Bustos, Rodrigo Fasce, Andres Castillo, Jorge Fernandez
EPI_ISL_1310630	Department of Pathology, University of Cambridge	COVID-19 Genomics UK (COG-UK) Consortium	Aminu S. Jahun, Yasmin Chaudhry, Iliana Georgana, Myra Hosmillo, Rhys Izuagbe, William L. Hamilton, Martin D. Curran, Surendra Parmar, Ian Goodfellow
EPI_ISL_1315322	LabPLUS	Institute of Environmental Science and Research (ESR)	Rachel Boyle, SallyAnn Harbison, Olivia Stroeven, Xiaoyun Ren, Matt Storey, Nikki Freed, Muhammad Faisal, Jing Wang, Hermes Perez, Anja Werno, Antje van der Linden, Arlo Upton, Chris Mansell, David Hammer, Dragana Drinkovic, Gary McAuliffe, Hana Sofia Andersson, James Ussher, Jill Sherwood, Josh Freeman, Julia Howard, Juliet Elvy, Mary DeAlmeida, Matt Blakiston, Matthew Rogers, Max Bloomfield, Michael Addidle, Michelle Balm, Sally Roberts, Sarah Jefferies, Sharmini Muttaiyah, Susan Morpeth, Susan Taylor, Timothy Blackmore, Vani Sathyendran, Veronica Playle, Virginia Hope, Erasmus Smit, Lauren Jelly, Olin Silander, Joep de Ligt
EPI_ISL_1315685, EPI_ISL_1316315, EPI_ISL_1316397, EPI_ISL_1316825, EPI_ISL_1344032, EPI_ISL_1344412, EPI_ISL_1344451, EPI_ISL_1345042, EPI_ISL_1345222, EPI_ISL_1345652, EPI_ISL_1345681, EPI_ISL_1345733, EPI_ISL_1345924, EPI_ISL_1345928, EPI_ISL_1377278, EPI_ISL_1378001, EPI_ISL_1378012, EPI_ISL_1378018, EPI_ISL_1378042, EPI_ISL_1378054, EPI_ISL_1378081, EPI_ISL_1378100, EPI_ISL_1378154, EPI_ISL_1378173, EPI_ISL_1378194, EPI_ISL_1378206, EPI_ISL_1378247, EPI_ISL_1378248, EPI_ISL_1378251, EPI_ISL_1378302, EPI_ISL_1378323, EPI_ISL_1389649, EPI_ISL_1389781, EPI_ISL_1389807, EPI_ISL_1389876, EPI_ISL_1389974, EPI_ISL_1389985, EPI_ISL_1390117, EPI_ISL_1390149, EPI_ISL_1390225, EPI_ISL_1390239, EPI_ISL_1390247, EPI_ISL_1390271, EPI_ISL_1390275, EPI_ISL_1390286, EPI_ISL_1390321, EPI_ISL_1390369, EPI_ISL_1410425, EPI_ISL_1410453, EPI_ISL_1410488, EPI_ISL_1410507, EPI_ISL_1410522, EPI_ISL_1410702, EPI_ISL_1410721, EPI_ISL_1412662, EPI_ISL_1412848, EPI_ISL_1413288			
see above	Lighthouse Lab in Glasgow	Wellcome Sanger Institute for the COVID-19 Genomics UK (COG-UK) Consortium	Harper VanSteenhouse, Yumi Kasai, David Gray, Carol Clugston, Anna Dominiczak and Alex Alderton, Roberto Amato, Jeffrey Barrett, Sonia Goncalves, Ewan Harrison, David K. Jackson, Ian Johnston, Dominic Kwiatkowski, Cordelia Langford, John Sillitoe on behalf of the Wellcome Sanger Institute COVID-19 Surveillance Team
EPI_ISL_1443680	Omics Sciences Laboratory	Omics Sciences Laboratory	Derly Andrade Molina, Rubén Armas González, Gabriel Morey León, Darlyn Amaya, Katheryn Sacheri Viteri, Emily Sulay Saltos Montalvo, Paula Juliana Gavilanes Jarrín, Juan Carlos Fernández Cadena
EPI_ISL_1470468	Genetica Molecular and Subdepartamento de Virologia ISP Chile	Instituto de Salud Publica de Chile	Javier Tognarelli, Karen Orostica, Barbara Parra, Loredana Arata, Jaime Lagos, Gisselle Barra, Patricia Bustos, Rodrigo Fasce, Andres Castillo, Jorge Fernandez
EPI_ISL_1491571	Institute for Urban Disease Control and Prevention	COVID-19 Network Investigations (CONI) Alliance	Elizabeth Batty, Wasun Chantratita, Thanat Chookajorn, Stefan Fernandez, Angkana Huang, Anthony R. Jones, Khajohn Joonlasak, Chonticha Klungtong, Theerarat Kochakarn, Namfon Kotanan, Krittikorn Kumpornsin, Duangkamon Loesbanluechai, Wudtichai Manasatienkij, Bhakbhoom Panthan, Ekawat Pasomsub, Kingkan Rakmanee, Insee Sensorn, Janjira Thaipadungpanit, Arporn Wangwiwatsin, Treewat Watthanachockchai, Kamolthip Atsawawaranunt, Prayuth Kaewmalang, Amornmas Kongklieng, Pukkaporn Parnwijitkul, Vichan Pawun
EPI_ISL_1494721	Area of Virology, Serology and Virology Division (SAViD), New South Wales Health Pathology Randwick	Virology Research Laboratory; Area of Virology, Serology and Virology Division (SAViD), New South Wales Health Pathology Randwick	Foster, C.; Au, J.; Ruiz Silva, M.; Deveson, I.; Bull, R.; Van Hal, S.; Rawlinson, W.
EPI_ISL_1506537, EPI_ISL_1506619, EPI_ISL_1506635, EPI_ISL_1506896, EPI_ISL_1506899, EPI_ISL_1506903, EPI_ISL_1506972, EPI_ISL_1506976, EPI_ISL_1506996, EPI_ISL_1507116, EPI_ISL_1507120, EPI_ISL_1507224, EPI_ISL_1507262, EPI_ISL_1507263, EPI_ISL_1507286, EPI_ISL_1507298, EPI_ISL_1507306, EPI_ISL_1507583, EPI_ISL_1507623, EPI_ISL_1507630, EPI_ISL_1507631, EPI_ISL_1507634, EPI_ISL_1507636, EPI_ISL_1507649, EPI_ISL_1507656, EPI_ISL_1507658, EPI_ISL_1507666, EPI_ISL_1507674, EPI_ISL_1507676, EPI_ISL_1507692, EPI_ISL_1507851, EPI_ISL_1507867, EPI_ISL_1507872, EPI_ISL_1507963, EPI_ISL_1507968, EPI_ISL_1507986, EPI_ISL_1508028, EPI_ISL_1508154, EPI_ISL_1508220, EPI_ISL_1508273, EPI_ISL_1508288, EPI_ISL_1508332, EPI_ISL_1508423, EPI_ISL_1508432, EPI_ISL_1508467, EPI_ISL_1508509, EPI_ISL_1508514, EPI_ISL_1508540, EPI_ISL_1508731, EPI_ISL_1508736, EPI_ISL_1508787, EPI_ISL_1508794			
see above	Lighthouse Lab in Glasgow	Wellcome Sanger Institute for the COVID-19 Genomics UK (COG-UK) Consortium	Harper VanSteenhouse, Yumi Kasai, David Gray, Carol Clugston, Anna Dominiczak and Alex Alderton, Roberto Amato, Jeffrey Barrett, Sonia Goncalves, Ewan Harrison, David K. Jackson, Ian Johnston, Dominic Kwiatkowski, Cordelia Langford, John Sillitoe on behalf of the Wellcome Sanger Institute COVID-19 Surveillance Team
EPI_ISL_1508993, EPI_ISL_1508997	PathWest Laboratory Medicine WA	PathWest Laboratory Medicine WA Microbial Surveillance Unit	PathWest Laboratory Medicine WA Microbial Surveillance Unit
EPI_ISL_1532815	Virology Unit, Institut Pasteur du Cambodge	Virology Unit, Institut Pasteur du Cambodge	Sokhoun Yann, Teyputita Ou, Leakhena Pum, Ly Sovann, Kraing Sidonn, Yi Sengdoeurn, Chin Savuth, Chau Darapheak, Veasna Duong, Erik A Karlsson
EPI_ISL_1537163, EPI_ISL_1537241, EPI_ISL_1537250, EPI_ISL_1537281, EPI_ISL_1537852, EPI_ISL_1538004, EPI_ISL_1538144	Lighthouse Lab in Glasgow	Wellcome Sanger Institute for the COVID-19 Genomics UK (COG-UK) Consortium	Harper VanSteenhouse, Yumi Kasai, David Gray, Carol Clugston, Anna Dominiczak and Alex Alderton, Roberto Amato, Jeffrey Barrett, Sonia Goncalves, Ewan Harrison, David K. Jackson, Ian Johnston, Dominic Kwiatkowski, Cordelia Langford, John Sillitoe on behalf of the Wellcome Sanger Institute COVID-19 Surveillance Team
EPI_ISL_1542497	Pandemic Response Lab - NYC	Pandemic Response Lab, R&D	Henry Lee, Michael Hammerling, Melissa Hopkins, Cybill del Castillo, Shinyoung Clair Kang, William Ward, Pradeep Bugga, Sol Rey, Dylan Law, Katharine

			Nelson, Haiping Hao, Jon Laurent
EPI_ISL_1544235, EPI_ISL_1544789, EPI_ISL_1544891, EPI_ISL_1545076	Lighthouse Lab in Glasgow	Wellcome Sanger Institute for the COVID-19 Genomics UK (COG-UK) Consortium	Harper VanSteenhouse, Yumi Kasai, David Gray, Carol Clugston, Anna Dominiczak and Alex Alderton, Roberto Amato, Jeffrey Barrett, Sonia Goncalves, Ewan Harrison, David K. Jackson, Ian Johnston, Dominic Kwiatkowski, Cordelia Langford, John Sillitoe on behalf of the Wellcome Sanger Institute COVID-19 Surveillance Team
EPI_ISL_1563660, EPI_ISL_1563665	PHV-FSS	PHV-FSS	Son Nguyen
EPI_ISL_1584561, EPI_ISL_1584639, EPI_ISL_1584652, EPI_ISL_1584745, EPI_ISL_1584768, EPI_ISL_1584784, EPI_ISL_1584970, EPI_ISL_1584996, EPI_ISL_1616240, EPI_ISL_1616268, EPI_ISL_1616331, EPI_ISL_1616375, EPI_ISL_1616379, EPI_ISL_1616586, EPI_ISL_1616590			
see above	Lighthouse Lab in Glasgow	Wellcome Sanger Institute for the COVID-19 Genomics UK (COG-UK) Consortium	Harper VanSteenhouse, Yumi Kasai, David Gray, Carol Clugston, Anna Dominiczak and Alex Alderton, Roberto Amato, Jeffrey Barrett, Sonia Goncalves, Ewan Harrison, David K. Jackson, Ian Johnston, Dominic Kwiatkowski, Cordelia Langford, John Sillitoe on behalf of the Wellcome Sanger Institute COVID-19 Surveillance Team
EPI_ISL_1621305, EPI_ISL_1621319	LabPLUS	Institute of Environmental Science and Research (ESR)	Rachel Boyle, SallyAnn Harbison, Olivia Stroeven, Xiaoyun Ren, Matt Storey, Nikki Freed, Muhammad Faisal, Jing Wang, Hermes Perez, Anja Werno, Antje van der Linden, Arlo Upton, Chris Mansell, David Hammer, Dragana Drinkovic, Gary McAuliffe, Hana Sofia Andersson, James Ussher, Jill Sherwood, Josh Freeman, Julia Howard, Juliet Elvy, Mary DeAlmeida, Matt Blakiston, Matthew Rogers, Max Bloomfield, Michael Addidle, Michelle Balm, Sally Roberts, Sarah Jefferies, Sharmini Muttaiyah, Susan Morpeth, Susan Taylor, Timothy Blackmore, Vani Sathyendran, Veronica Playle, Virginia Hope, Erasmus Smit, Lauren Jelly, Olin Silander, Joep de Ligt
EPI_ISL_1634428	National Public Health Laboratory, National Centre for Infectious Diseases	National Public Health Laboratory, National Centre for Infectious Diseases	Tze Minn Mak, Zhenyang Zhou, Grace Jie Yin Ngan, Royce Ang, Lin Cui, Raymond Tzer Pin Lin
EPI_ISL_1634703, EPI_ISL_1634865, EPI_ISL_1634943, EPI_ISL_1635027, EPI_ISL_1635034, EPI_ISL_1635113, EPI_ISL_1635118, EPI_ISL_1635168, EPI_ISL_1635353	Lighthouse Lab in Glasgow	Wellcome Sanger Institute for the COVID-19 Genomics UK (COG-UK) Consortium	Harper VanSteenhouse, Yumi Kasai, David Gray, Carol Clugston, Anna Dominiczak and Alex Alderton, Roberto Amato, Jeffrey Barrett, Sonia Goncalves, Ewan Harrison, David K. Jackson, Ian Johnston, Dominic Kwiatkowski, Cordelia Langford, John Sillitoe on behalf of the Wellcome Sanger Institute COVID-19 Surveillance Team
EPI_ISL_1647662	Public Health Authority of the Slovak Republic	Laboratory of Genomics and Bioinformatics, Comenius University Science Park	Tatiana Sedláková, Diana Rusáková, Miroslav Böhmer, Anna Giová, Jaroslav Budiš, Tomáš Szemes
EPI_ISL_1662457	Immunogenomics lab, Institute of Life Sciences, Bhubaneswar	Institute of Life Sciences - INSACOG	Sunil K. Raghav, Safal Walia, Arup Ghosh, Atimukta Jha, Amol M. Kanampalliwar, Shifu Aggarwal, Rupesh Dash, Rajeeb Swain, Punit Prasad, INSACOG Consortium, Ajay Parida
EPI_ISL_1663384	NCCS, Pune	Institute of Life Sciences - INSACOG	Sunil K. Raghav, Safal Walia, Arup Ghosh, Atimukta Jha, Amol M. Kanampalliwar, Shifu Aggarwal, Rupesh Dash, Rajeeb Swain, Punit Prasad, INSACOG Consortium, Ajay Parida
EPI_ISL_1663501	Veer Surendra Sai Institute of Medical Sciences and Research, Burla, Sambalpur	Institute of Life Sciences - INSACOG	Sunil K. Raghav, Safal Walia, Arup Ghosh, Atimukta Jha, Amol M. Kanampalliwar, Shifu Aggarwal, Rupesh Dash, Rajeeb Swain, Punit Prasad, INSACOG Consortium, Ajay Parida
EPI_ISL_1672975, EPI_ISL_1672980, EPI_ISL_1672985, EPI_ISL_1672989, EPI_ISL_1672992, EPI_ISL_1673110, EPI_ISL_1673143, EPI_ISL_1673229	Lighthouse Lab in Glasgow	Wellcome Sanger Institute for the COVID-19 Genomics UK (COG-UK) Consortium	Harper VanSteenhouse, Yumi Kasai, David Gray, Carol Clugston, Anna Dominiczak and Alex Alderton, Roberto Amato, Jeffrey Barrett, Sonia Goncalves, Ewan Harrison, David K. Jackson, Ian Johnston, Dominic Kwiatkowski, Cordelia Langford, John Sillitoe on behalf of the Wellcome Sanger Institute COVID-19 Surveillance Team
EPI_ISL_1684014	Laboratory Corporation of America	Centers for Disease Control and Prevention Division of Viral Diseases, Pathogen Discovery	Dakota Howard, Dhwani Batra, Peter W. Cook, Kara Moser, Adrian Paskey, Jason Caravas, Benjamin Rambo-Martin, Shatavia Morrison, Christopher Gulvick, Scott Sammons, Yvette Unoarumhi, Darlene Wagner, Matthew Schmerer, Minoo Agarwal, Eyad Almasri, Debbie Boles, Ayla Burns, Nuthawin Charoensri, Oren Cohen, Susan Countryman, Mary Ann Cristobal, Bobbi Croy, Suzanne Dale, Hrushikesh Deshmukh, Amanda Douglas, Vincent Drouillon, Marcia Eisenberg, Howard Engler, Rama Ghatti, Prashant Gupta, Susan Hicks, Jake Humphrey, Lax Iyer, Manoj Jain, Mohan Kolli, Brian Krueger, Tim Kuphal, Stanley Letovsky, Michael Levandoski, Craig Lukasik, Jonathan Meltzer, Brian Norvell, Mindy Nye, Scott Parker, Christos Petropoulos, John Pruitt, Steven Ragan, Scott Ryan, Mike Sapeta, Jana Schroth, Suresh Babu Selvaraju, Goran Stevovic, Amanda Suchanek, Andrea Throop, Lyndon Tilson, Thomas Urban, Joe Voshell, Kimberly Wagner, Jonathan Williams, Mary Williamson, Qian Zeng, Tricia Zwiefelhofer, Clinton R. Paden, Duncan MacCannell
EPI_ISL_1695988	CH. CHU DE REIMS	Department of Virology, Henri Mondor University Hospital, Assistance Publique Hôpitaux de Paris, Université Paris-Est Créteil, INSERM U955	Christophe Rodriguez, Slim Fourati, Vanessa Demontant, Guillaume Gricourt, Melissa N'Debi, Alexandre Soulier, Elisabeth Trawinski, Jean-Michel Pawlotsky
EPI_ISL_1697280	New South Wales Health Pathology Royal Prince Alfred Hospital	Microbiology RPAH	Foster, C.; Au, J.; Ruiz Silva, M.; Deveson, I.; Bull, R.; Van Hal, S.; Rawlinson, W.
EPI_ISL_1698208, EPI_ISL_1698780, EPI_ISL_1698895, EPI_ISL_1698917	Lighthouse Lab in Milton Keynes	Wellcome Sanger Institute for the COVID-19 Genomics UK (COG-UK) Consortium	The Lighthouse Lab in Milton Keynes and Alex Alderton, Roberto Amato, Jeffrey Barrett, Sonia Goncalves, Ewan Harrison, David K. Jackson, Ian Johnston, Dominic Kwiatkowski, Cordelia Langford, John Sillitoe on behalf of the Wellcome Sanger Institute COVID-19 Surveillance Team
EPI_ISL_1715389	Nigerian Centre for Disease Control (NCDC)	African Centre of Excellence for Genomics of Infectious Diseases, Redeemer's University	Olawoye, I.B. et al
EPI_ISL_1741290	Lighthouse Lab in Milton Keynes	Wellcome Sanger Institute for the COVID-19 Genomics UK (COG-UK) Consortium	The Lighthouse Lab in Milton Keynes and Alex Alderton, Roberto Amato, Jeffrey Barrett, Sonia Goncalves, Ewan Harrison, David K. Jackson, Ian Johnston, Dominic Kwiatkowski, Cordelia Langford, John Sillitoe on behalf of the Wellcome Sanger Institute COVID-19 Surveillance Team
EPI_ISL_1741562, EPI_ISL_1741577, EPI_ISL_1741658, EPI_ISL_1741667, EPI_ISL_1741707, EPI_ISL_1741731, EPI_ISL_1741734, EPI_ISL_1741744, EPI_ISL_1741745, EPI_ISL_1741756, EPI_ISL_1741888, EPI_ISL_1741938, EPI_ISL_1741972			
see above	Lighthouse Lab in Glasgow	Wellcome Sanger Institute for the COVID-19 Genomics UK (COG-UK) Consortium	Harper VanSteenhouse, Yumi Kasai, David Gray, Carol Clugston, Anna Dominiczak and Alex Alderton, Roberto Amato, Jeffrey Barrett, Sonia Goncalves, Ewan Harrison, David K. Jackson, Ian Johnston, Dominic Kwiatkowski, Cordelia Langford, John Sillitoe on behalf of the Wellcome Sanger Institute COVID-19 Surveillance Team
EPI_ISL_1741991	Lighthouse Lab in Milton Keynes	Wellcome Sanger Institute for the COVID-19 Genomics UK (COG-UK) Consortium	The Lighthouse Lab in Milton Keynes and Alex Alderton, Roberto Amato, Jeffrey Barrett, Sonia Goncalves, Ewan Harrison, David K. Jackson, Ian Johnston, Dominic Kwiatkowski, Cordelia Langford, John Sillitoe on behalf of the Wellcome Sanger Institute COVID-19 Surveillance Team
EPI_ISL_1742065, EPI_ISL_1742219, EPI_ISL_1742249	Lighthouse Lab in Glasgow	Wellcome Sanger Institute for the COVID-19 Genomics UK (COG-UK) Consortium	Harper VanSteenhouse, Yumi Kasai, David Gray, Carol Clugston, Anna Dominiczak and Alex Alderton, Roberto Amato, Jeffrey Barrett, Sonia Goncalves, Ewan Harrison, David K. Jackson, Ian Johnston, Dominic Kwiatkowski, Cordelia Langford, John Sillitoe on behalf of the Wellcome Sanger Institute COVID-19 Surveillance Team
EPI_ISL_1789287	UW Virology Lab	UW Virology Lab	Pavitra Roychoudhury, Hong Xie, Lasata Shrestha, Tien V. Nguyen, Shah Mohamed Bakhash, Michelle Lin, Noah R. Baker, Sean Ellis, Meei-Li Huang, Keith R Jerome, Alexander Greninger
EPI_ISL_1789542	WHO National Influenza Centre Russian Federation	WHO National Influenza Centre Russian Federation	Andrey Komissarov, Artem Fadeev, Kseniya Komissarova, Oula Mansour, Mikhail Bakaev, Tamila Musaeva, Maria Timofeeva, Veronika Eder, Maria Pisareva, Daria Danilenko, Ksenia Safina, Elena Nabieva, Georgii Bazykin, Dmitry Lioznov
EPI_ISL_1816917, EPI_ISL_1817558	PathWest Laboratory Medicine WA	PathWest Laboratory Medicine WA Microbial Surveillance Unit	PathWest Laboratory Medicine WA Microbial Surveillance Unit
EPI_ISL_1823177	Biolab Diagnostic Laboratories	Biolab Diagnostic Laboratories	Issa Abu-Dayyeh, Ahmad Tibi, Lama Hussein, Shayma Ali, Badia Saddedin, Eiad Atwa, Amid Abdelnour
EPI_ISL_1827951	Australian Infectious Disease Research Centre, School of Chemistry and Molecular Biosciences, The University of Queensland	Australian Infectious Disease Research Centre, School of Chemistry and Molecular Biosciences, The University of Queensland	Parry,R.H. and Khromykh,A.
EPI_ISL_1838015	The National Centre for Cell Science	CSIR-Centre for Cellular and Molecular Biology-INSACOG	Dhiraj Paul, Mohak P Gujare, Shivang P. Bhanushali, Mitali Inamdar, Sonal Manik Chavan, Manoj Kumar Bhat, Ajay Pillai, INSACOG Consortium team, Yogesh Shouche,Payel Mukherjee,Lamuk Zaveri,Tulasi Nagabandi, Ara Sreenivas,Valli Nagalakshmi Undamatla,Shreekant Verma, Amareshwar Vodapalli ,Blessy B John,Viswagithe S L,B Himasri,Onkar Kulkarni,Sofia Banu,Archana Bharadwaj Siva,Sharath Chandra Thota,Karthik Bharadwaj Tallapaka,Rakesh K Mishra,Divya Tej Sowpati

EPI_ISL_1841252, EPI_ISL_1841383	ICMR-National Institute of Virology - INSACOG	NIV Influenza	Dr. Varsha Potdar
EPI_ISL_1846077	Eurofins LifeCodexx GmbH	Robert Koch Institute	unknown
EPI_ISL_1904454	South Eastern Area Laboratory Services (SEALS)	NSW Health Pathology - Institute of Clinical Pathology and Medical Research; Westmead Hospital; University of Sydney	CIDM-PH et al.
EPI_ISL_1904852	LabPLUS	Institute of Environmental Science and Research (ESR)	Rachel Boyle, SallyAnn Harbison, Olivia Stroeven, Xiaoyun Ren, Matt Storey, Nikki Freed, Muhammad Faisal, Jing Wang, Hermes Perez, Anja Werno, Antje van der Linden, Arlo Upton, Chris Mansell, David Hammer, Dragana Drinkovic, Gary McAuliffe, Hana Sofia Andersson, James Ussher, Jill Sherwood, Josh Freeman, Julia Howard, Juliet Elvy, Mary DeAlmeida, Matt Blakiston, Matthew Rogers, Max Bloomfield, Michael Addidle, Michelle Balm, Sally Roberts, Sarah Jefferies, Sharmini Muttaiyah, Susan Morpeth, Susan Taylor, Timothy Blackmore, Vani Sathyendran, Veronica Playle, Virginia Hope, Erasmus Smit, Lauren Jelly, Olin Silander, Joep de Ligt
EPI_ISL_1911187, EPI_ISL_1911189	Area of Virology, Serology and Virology Division (SAViD), New South Wales Health Pathology Randwick	Virology Research Laboratory; Area of Virology, Serology and Virology Division (SAViD), New South Wales Health Pathology Randwick	Foster, C.; Au, J.; Ruiz Silva, M.; Deveson, I.; Bull, R.; Van Hal, S.; Rawlinson, W.
EPI_ISL_1911195, EPI_ISL_1911196, EPI_ISL_1911197, EPI_ISL_1911250	Zhejiang Provincial Center for Disease Control and Prevention Zhoushan Center for Disease Prevention and Contol	Zhejiang Province Center of Disease Control and prevention	Yanjun Zhang, Bing WU, Hongling Wang
EPI_ISL_1913206, EPI_ISL_1913208, EPI_ISL_1913209	Victorian Infectious Diseases Reference Laboratory (VIDRL)	VIDRL and MDU-PHL	Caly L., Seemann T., Sait, M.L., Druce J., Sherry, N.L.
EPI_ISL_1914668	PathWest Laboratory Medicine WA	PathWest Laboratory Medicine WA Microbial Surveillance Unit	PathWest Laboratory Medicine WA Microbial Surveillance Unit
EPI_ISL_1919423	WHO National Influenza Centre Russian Federation	WHO National Influenza Centre Russian Federation	Andrey Komissarov, Artem Fadeev, Kseniya Komissarova, Oula Masour, Kirill Varchenko, Mikhail Bakaev, Tamila Musaeva, Maria Timofeeva, Veronika Eder, Maria Pisareva, Nikita Yolshin, Daria Danilenko, Ksenia Safina, Elena Nabieva, Georgii Bazykin, Dmitry Lioznov
EPI_ISL_1919757	Ospedale Santa Caterina Novella	Istituto Zooprofilattico Sperimentale della Puglia e della Basilicata	Parisi A., Bianco A., Capozzi L., Del Sambro L., Simone D., Difato L., Bruno A.
EPI_ISL_1928467	ICMR-National Institute of Virology - INSACOG	NIV Influenza	Dr. Varsha Potdar
EPI_ISL_1934669	Division of Emerging Infectious Diseases, Bureau of Infectious Diseases Diagnosis Control, Korea Disease Control and Prevention Agency	Division of Emerging Infectious Diseases, Bureau of Infectious Diseases Diagnosis Control, Korea Disease Control and Prevention Agency	Ae Kyung Park, Il-Hwan Kim, Heui Man Kim, Jeong-Min Kim, Jeong-Ah Kim, Chae Young Lee, Jin Sun No, Eun-Jin Kim
EPI_ISL_1939905	NCCS	inStem NCBS - INSACOG	Uma Ramakrishnan Dasaradhi Palakodeti Aswin SaiNarain
EPI_ISL_1970348	Chongqing International Travel Health Care Center	Chongqing Municipal Center for Disease Control and Prevention	Sheng Ye, Shuang Chen, Haiyan Wen, Zhen Yu , Lan Zhou, Hua Ling, Dong Wang, Yun Tang, Mingyue Wang, Zhangping Tan, Wenge Tang, Rong Rong
EPI_ISL_1970564	MRC/UVRI & LSHTM Uganda Research Unit	MRC/UVRI & LSHTM Uganda Research Unit	Matthew Cotten, Dan Lule Bugembe, My V.T. Phan, Isaac Sseeewanyana, Patrick Semanda, Susan Nabadda, Pontiano Kaleebu
EPI_ISL_1972356	Sungai Buloh Hospital	Institute for Medical Research, Infectious Disease Research Centre, National Institutes of Health, Ministry of Health Malaysia	Suppiah J, Kamel K, Mohd Zawawi Z, Azizan MA, Ramly N, Robert F, Thayan R
EPI_ISL_1972908, EPI_ISL_1972909, EPI_ISL_1972911, EPI_ISL_1972912	PathWest Laboratory Medicine WA	PathWest Laboratory Medicine WA Microbial Surveillance Unit	PathWest Laboratory Medicine WA Microbial Surveillance Unit
EPI_ISL_1990741	Helix/Illumina	Centers for Disease Control and Prevention Division of Viral Diseases, Pathogen Discovery	Dakota Howard, Dhwani Batra, Peter W. Cook, Kara Moser, Adrian Paskey, Jason Caravas, Benjamin Rambo-Martin, Shatavia Morrison, Christopher Gulvick, Scott Sammons, Yvette Unoarumhi, Darlene Wagner, Matthew Schmerer, Eileen de Feo, Jan Antico, Christine Tran, Matthew Tolentino, Shannon Wickline, Kim Gietzen, Brad Sickler, Jingtao Liu, Eric Allen, Phil Febbo, Nicole L. Washington, Simon White, Geraint Levan, Kelly Schiabor Barrett, Elizabeth Cirulli, Alexandre Bolze, Ary Ascencio, Charlotte Rivera-Garcia, Ryan Cho, Jason Nguyen, Sherry Wang, Jimmy Ramirez, Tyler Cassens, Efren Sandoval, Magnus Isaksson, William Lee, David Becker, Marc Laurent, James Lu, Clinton R. Paden, Duncan MacCannell
EPI_ISL_1999092	Aegis Sciences Corporation	Centers for Disease Control and Prevention Division of Viral Diseases, Pathogen Discovery	Dakota Howard, Dhwani Batra, Peter W. Cook, Kara Moser, Adrian Paskey, Jason Caravas, Benjamin Rambo-Martin, Shatavia Morrison, Christopher Gulvick, Scott Sammons, Yvette Unoarumhi, Darlene Wagner, Matthew Schmerer, Cyndi Clark, Patrick Campbell, Rob Case, Vikramsinha Ghorpade, Holly Houdeshell, Ola Kvalvaag, Dillon Nall, Ethan Sanders, Alec Vest, Shaun Westlund, Matthew Hardison, Clinton R. Paden, Duncan MacCannell
EPI_ISL_2001060	PHV-FSS	PHV-FSS	Son Nguyen
EPI_ISL_2008369	Massachusetts State Public Health Laboratory	Massachusetts State Public Health Laboratory	Andrew Lang, Timelia Fink, Glen Gallagher, Sandra Smole
EPI_ISL_2020946	Urban Public Health Centre (UPHC), SECTOR-2, Gandhinagar	Gujarat Biotechnology Research Centre	Bhumika Prajapati,Twinkle Soni,Sonal Sharma ,Zuber Saiyed,Ramesh Pandit,Janvi Raval,Zarna Patel,Nitin Savaliya,Dinesh Kumar,Bhargav Waghela,Tejendra Purohit ,Nilen Patel ,Amit Kanani,Kalpesh Goswami,Amrutlal Patel,Umang Mishra,Nitesh Shah,Chaitanya Joshi,Madhvi Joshi
EPI_ISL_2034589	Swedish national genomic surveillance program of SARS-CoV-2	The Public Health Agency of Sweden	Maximilian Riess, Maria Lind Karlberg, Alma Brolund, Swedish national genomic surveillance program of SARS-CoV-2
EPI_ISL_2039336, EPI_ISL_2040357, EPI_ISL_2043219	Aegis Sciences Corporation	Centers for Disease Control and Prevention Division of Viral Diseases, Pathogen Discovery	Dakota Howard, Dhwani Batra, Peter W. Cook, Kara Moser, Adrian Paskey, Jason Caravas, Benjamin Rambo-Martin, Shatavia Morrison, Christopher Gulvick, Scott Sammons, Yvette Unoarumhi, Darlene Wagner, Matthew Schmerer, Cyndi Clark, Patrick Campbell, Rob Case, Vikramsinha Ghorpade, Holly Houdeshell, Ola Kvalvaag, Dillon Nall, Ethan Sanders, Alec Vest, Shaun Westlund, Matthew Hardison, Clinton R. Paden, Duncan MacCannell
EPI_ISL_2081529	PathWest Laboratory Medicine WA	PathWest Laboratory Medicine WA Microbial Surveillance Unit	PathWest Laboratory Medicine WA Microbial Surveillance Unit
EPI_ISL_2089684, EPI_ISL_2089942, EPI_ISL_2089944, EPI_ISL_2090122	Aegis Sciences Corporation	Centers for Disease Control and Prevention Division of Viral Diseases, Pathogen Discovery	Dakota Howard, Dhwani Batra, Peter W. Cook, Kara Moser, Adrian Paskey, Jason Caravas, Benjamin Rambo-Martin, Shatavia Morrison, Christopher Gulvick, Scott Sammons, Yvette Unoarumhi, Darlene Wagner, Matthew Schmerer, Cyndi Clark, Patrick Campbell, Rob Case, Vikramsinha Ghorpade, Holly Houdeshell, Ola Kvalvaag, Dillon Nall, Ethan Sanders, Alec Vest, Shaun Westlund, Matthew Hardison, Clinton R. Paden, Duncan MacCannell
EPI_ISL_2091086	University Hospitals Translational Laboratory (UHTL), University Hospitals	University Hospitals Translational Laboratory (UHTL), University Hospitals	Sadri,N., Alouani,D., Song,X.
EPI_ISL_2098723	PathWest Laboratory Medicine WA	PathWest Laboratory Medicine WA Microbial Surveillance Unit	PathWest Laboratory Medicine WA Microbial Surveillance Unit
EPI_ISL_2100246	Clinical Hospital of Infectious and Tropical Diseases "Dr. Victor Babes"	Cantacuzino Institute Virology	Sorin Dinu,Carmen Cherciu, Mihaela Oprea, Mihaela Lazar
EPI_ISL_2103201, EPI_ISL_2103202	LabPLUS	Institute of Environmental Science and Research (ESR)	Rachel Boyle, SallyAnn Harbison, Olivia Stroeven, Xiaoyun Ren, Matt Storey, Nikki Freed, Muhammad Faisal, Jing Wang, Hermes Perez, Anja Werno, Antje van der Linden, Arlo Upton, Chris Mansell, David Hammer, Dragana Drinkovic, Gary McAuliffe, Hana Sofia Andersson, James Ussher, Jill Sherwood, Josh Freeman, Julia Howard, Juliet Elvy, Mary DeAlmeida, Matt Blakiston, Matthew Rogers, Max Bloomfield, Michael Addidle, Michelle Balm, Sally Roberts, Sarah Jefferies, Sharmini Muttaiyah, Susan Morpeth, Susan Taylor, Timothy Blackmore, Vani Sathyendran, Veronica Playle, Virginia Hope, Erasmus Smit, Lauren Jelly, Olin Silander, Joep de Ligt
EPI_ISL_2105672	Biolab Diagnostic Laboratories	Biolab Diagnostic Laboratories	Issa Abu-Dayyeh, Ahmad Tibi, Lama Hussein, Shayma Ali, Badia Saddedin, Eiad Atwa, Amid Abdelnour
EPI_ISL_2107020, EPI_ISL_2107054	National Centre For Cell Science - INSACOG	National Centre For Cell Science	Dhiraj Paul, Shivang P. Bhanushali, Mohak P Gujare, Mitali Inamdar, Manoj Kumar Bhat, Ajay Pillai, INSACOG Consortium team, Yogesh Shouche
EPI_ISL_2107443, EPI_ISL_2107529	New South Wales Health Pathology Royal Prince Alfred Hospital	Microbiology RPAH	Foster, C.; Au, J.; Ruiz Silva, M.; Deveson, I.; Bull, R.; Van Hal, S.; Rawlinson, W.
EPI_ISL_2123724	Eurofins LifeCodexx GmbH	Robert Koch Institute	unknown
EPI_ISL_2125129	Labor Dr. Spranger	Robert Koch Institute	unknown

EPI_ISL_2131788	SARS-CoV-2 testing team, National Institute of Infectious Diseases	Pathogen Genomics Center, National Institute of Infectious Diseases	Tsuyoshi Sekizuka, Kentaro Itokawa, Rina Tanaka, Masanori Hashino, Nozomu Hanaoka, Masumichi Saito, Naomi Nojiri, Hazuka Y Furihata, Sana Uchikoba, Tsuguto Fujimoto, Makoto Kuroda
EPI_ISL_2135843, EPI_ISL_2135844, EPI_ISL_2135846	Viral Respiratory Lab, National Institute for Biomedical Research (INRB)	Pathogen Sequencing Lab, National Institute for Biomedical Research (INRB)	Placide Mbala-Kingebeni, Edith Nkwembe, Eddy Kinganda-Lusamaki, Amuri Aziza, Francisca Muyembe Mawete, Emmanuel Lokilo Lofiko, Jean Claude Makangara, Catherine Pratt, Matthias Pauthner, Josh Quick, Allison Black, James Hadfield, Trevor Bedford, Ian Goodfellow, Andrew Rambaut, Nick Loman, Kristian Andersen, Michael Wiley, Steve Ahuka-Mundeke, Jean-Jacques Muyembe Tamfum
EPI_ISL_2145882, EPI_ISL_2145895, EPI_ISL_2146150, EPI_ISL_2146944, EPI_ISL_2146965, EPI_ISL_2147487, EPI_ISL_2147930, EPI_ISL_2148121, EPI_ISL_2148833, EPI_ISL_2148878, EPI_ISL_2149227, EPI_ISL_2149713, EPI_ISL_2150223, EPI_ISL_2150247			
see above	Aegis Sciences Corporation	Centers for Disease Control and Prevention Division of Viral Diseases, Pathogen Discovery	Dakota Howard, Dhwani Batra, Peter W. Cook, Kara Moser, Adrian Paskey, Jason Caravas, Benjamin Rambo-Martin, Shatavia Morrison, Christopher Gulvick, Scott Sammons, Yvette Unoarumhi, Darlene Wagner, Matthew Schmerer, Cyndi Clark, Patrick Campbell, Rob Case, Vikramsinha Ghorpade, Holly Houdeshell, Ola Kvalvaag, Dillon Nall, Ethan Sanders, Alec Vest, Shaun Westlund, Matthew Hardison, Clinton R. Paden, Duncan MacCannell
EPI_ISL_2158597	Laboratório de Biologia Molecular Jean Piaget	MRCG at LSHTM, Genomics lab	Aladje Balde, Abdul Karim Sesay, Abdoulie Kanteh, Bakary Sanyang, Simão Tchuda Bióté, Rui Inndi, Adul Candé, Faatu Cassama, Milanca Agostinho Cá, Rei José Pereira, Erica Luis Maria Magalhães, Aicha Balde, Bubacar Delgado Pinto Embaló, Edmira Maria da Costa, Paulina Joãozinho da Costa Jarra Manneh, Mariama Kujabi, Dabiri Damilari, Sainabou Laye Ndure
EPI_ISL_2158913	Aegis Sciences Corporation	Centers for Disease Control and Prevention Division of Viral Diseases, Pathogen Discovery	Dakota Howard, Dhwani Batra, Peter W. Cook, Kara Moser, Adrian Paskey, Jason Caravas, Benjamin Rambo-Martin, Shatavia Morrison, Christopher Gulvick, Scott Sammons, Yvette Unoarumhi, Darlene Wagner, Matthew Schmerer, Cyndi Clark, Patrick Campbell, Rob Case, Vikramsinha Ghorpade, Holly Houdeshell, Ola Kvalvaag, Dillon Nall, Ethan Sanders, Alec Vest, Shaun Westlund, Matthew Hardison, Clinton R. Paden, Duncan MacCannell
EPI_ISL_2159811	Helix/Illumina	Centers for Disease Control and Prevention Division of Viral Diseases, Pathogen Discovery	Dakota Howard, Dhwani Batra, Peter W. Cook, Kara Moser, Adrian Paskey, Jason Caravas, Benjamin Rambo-Martin, Shatavia Morrison, Christopher Gulvick, Scott Sammons, Yvette Unoarumhi, Darlene Wagner, Matthew Schmerer, Eileen de Feo, Jan Antico, Christine Tran, Matthew Tolentino, Shannon Wickline, Kim Gietzen, Brad Sickler, Jingtao Liu, Eric Allen, Phil Febbo, Nicole L. Washington, Simon White, Geraint Levan, Kelly Schiabor Barrett, Elizabeth Cirulli, Alexandre Bolze, Ary Ascencio, Charlotte Rivera-Garcia, Ryan Cho, Jason Nguyen, Sherry Wang, Jimmy Ramirez, Tyler Cassens, Efren Sandoval, Magnus Isaksson, William Lee, David Becker, Marc Laurent, James Lu, Clinton R. Paden, Duncan MacCannell
EPI_ISL_2161784	NL-Dr. Leonard A. Miller Centre for Health Services	National Microbiology Laboratory (NML)	Anna Majer, Shari Tyson, Grace Seo, Philip Mabon, Elsie Grudeski, Rhiannon Huzarewich, Russell Mandes, Anneliese Landgraff, Jennifer Tanner, Natalie Knox, Morag Graham, Gary Van Domselaar, Robert Needle, Yang Yu, Adel Malek, Laura Gilbert, George Zahariadis, Nathalie Bastien, Yan Li, Timothy Booth, Darian Hole, Madison Chapel, Kirsten Biggar, Kerri Smith, CanCOGeN's metadata curation team, Public Health Agency of Canada CanCOGeN team
EPI_ISL_2171267	Dhulikhel Hospital, Kathmandu University Hospital	Molecular and Genomics Research Lab, Dhulikhel Hospital, Kathmandu University Hospital	Rajeev Shrestha, Nishan Katuwal, Navin Adhikari, Dipesh Tamrakar, Meghnath Dhimal, Pradip Gyanwali, Saroj Bhattarai, Surendra Kumar Madhup
EPI_ISL_2187722	HLAGYN - Laboratorio de Imunologia de Transplantes de Goias	HLAGYN - Laboratorio de Imunologia de Transplantes de Goias	Fernando Antonio Vinhal dos Santos, Erika Lopes Rocha Batista, Alessandro Leonardo Alvares Magalhaes, Frederico Rodrigues Vinhal, Sabrina Sara Moreira Duarte, Lucas Carlos Gomes Pereira, Daniel Ferreira de Sousa
EPI_ISL_2189756	ICMR-National Institute of Virology	NCDC Delhi, Biotechnology Division	Meena Datta,V.Vipat,S.Jadhav,Priyanka Singh,U.Saha ,M.Das,Uma Sharma,G.Divekar,T.Sanjeevi,Manoj K Singh,S Jadhav,Radhakrishnan V. S,A Walimbe ,Robin Marwal,V Malik,Mahesh S Dhar,K.Iyengar,Kalaiarasan Ponnusamy,K P.Shinde,Hemlata Lall,K.Korabu,Z. Sayyed,Hema Gogia, S.Shelkande,R.Verma,Preeti Madan,S.Bhorekar,B.Apoorva,Sandhya Kabra,A.Titkare,H.Kengale,Sujeet K Singh,V.Autude,A.Awhale,M L Choudhary,Partha Rakshit
EPI_ISL_2190106	National Institute of Infectious Diseases-Prof. Dr. Matei Bals Molecular Diagnostics Laboratory	National Institute of Infectious Diseases-Prof. Dr. Matei Bals Molecular Diagnostics Laboratory	Corina Casangiu, Leontina Banica, Marius Surleac, Ovidiu Vlaicu, Andreea Tudor, Simona Paraschiv, Dan Otelea
EPI_ISL_2199515, EPI_ISL_2199525	Lighthouse Lab in Alderley Park	Wellcome Sanger Institute for the COVID-19 Genomics UK (COG-UK) Consortium	Jacquelyn Wynn, Mairead Hyland, The Lighthouse Lab in Alderley Park and Alex Alderton, Roberto Amato, Jeffrey Barrett, Sonia Goncalves, Ewan Harrison, David K. Jackson, Ian Johnston, Dominic Kwiatkowski, Cordelia Langford, John Sillitoe on behalf of the Wellcome Sanger Institute COVID-19 Surveillance Team
EPI_ISL_2207016	Nebraska Public Health Laboratory	NPHL COVID-19 Response Team	NPHL COVID-19 Response Team
EPI_ISL_2227204	COVID-19 National Reference Laboratoty, Pasteur Institute of Iran	Genetics Research Center, University of Social Welfare and Rehabilitation Sciences	Zohreh Fattahi, Marzieh Mohseni, Kimia Kahrizi, Mahsa Tavakoli,Tahmineh Jalali, Mohammad Hassan Pouriayevali, Mostafa Salehi-Vaziri Hossein Najmbadi.
EPI_ISL_2227268	COVID-19 National Reference Laboratoty, Pasteur Institute of Iran	Genetics Research Center, University of Social Welfare and Rehabilitation Sciences	Zohreh Fattahi, Marzieh Mohseni, Kimia Kahrizi, Mahsa Tavakoli,Tahmineh Jalali, Mohammad Hassan Pouriayevali, Mostafa Salehi-Vaziri, Hossein Najmbadi.
EPI_ISL_2227269	COVID-19 National Reference Laboratoty, Pasteur Institute of Iran	Genetics Research Center, University of Social Welfare and Rehabilitation Sciences	Zohreh Fattahi, Marzieh Mohseni, Kimia Kahrizi, Mahsa Tavakoli,Tahmineh Jalali, Mohammad Hassan Pouriayevali, Mostafa Salehi-Vaziri, Hossein Najmbadi*.
EPI_ISL_2227270	COVID-19 National Reference Laboratoty, Pasteur Institute of Iran.	Genetics Research Center, University of Social Welfare and Rehabilitation Sciences	Zohreh Fattahi, Marzieh Mohseni, Kimia Kahrizi, Mahsa Tavakoli,Tahmineh Jalali, Mohammad Hassan Pouriayevali, Mostafa Salehi-Vaziri, Hossein Najmbadi.
EPI_ISL_2227271	COVID-19 National Reference Laboratory, Pasteur Institute of Iran.	Genetics Research Center, University of Social Welfare and Rehabilitation Sciences	Zohreh Fattahi, Marzieh Mohseni, Kimia Kahrizi, Mahsa Tavakoli,Tahmineh Jalali, Mohammad Hassan Pouriayevali, Mostafa Salehi-Vaziri, Hossein Najmbadi.
EPI_ISL_2227272	COVID-19 National Reference Laboratory, Pasteur Institute of Iran	Genetics Research Center, University of Social Welfare and Rehabilitation Sciences	Zohreh Fattahi, Marzieh Mohseni, Kimia Kahrizi, Mahsa Tavakoli,Tahmineh Jalali, Mohammad Hassan Pouriayevali, Mostafa Salehi-Vaziri, Hossein Najmbadi.
EPI_ISL_2230722	Southern Nevada Public Health Laboratory	Southern Nevada Public Health Laboratory	Michael Picker
EPI_ISL_2231758	AP SSO	CSIR-Centre for Cellular and Molecular Biology-INSACOG	Tulasi Nagabandi, Lamuk Zaveri,Ara Sreenivas,Shreekant Verma, Amareshwar Vodapalli ,Blessy B John,Viswagithe S L,B Himasri,Valli Nagalakshmi Undamatla,Payel Mukherjee,Sofia Banu,Archana Bharadwaj Siva,Sharath Chandra Thota,Karthik Bharadwaj Tallapaka,Rakesh K Mishra,Divya Tej Sowpati
EPI_ISL_2233099	Kantor Kesehatan Pelabuhan Kelas II Cilacap	National Institute of Health Research and Development	Subangkit, Hana Apsari Pawestri, Hartanti Dian Ikawati, Kartika Dewi Puspa, Arie Ardiansyah Nugraha, Triyani Soekarso, Krisna Pangesti, Nelly Puspandari, Vivi Setiawaty
EPI_ISL_2233382	Hospital Labuan	Institute for Medical Research, Infectious Disease Research Centre, National Institutes of Health, Ministry of Health Malaysia	Suppiah J, Kamel K, Mohd Zawawi Z, Azizan MA, Ramly N, Robert F, Thayan R
EPI_ISL_2249015	unknown	Instituto Nacional de Saude (INSA)	Borges et al
EPI_ISL_2250197, EPI_ISL_2250203, EPI_ISL_2250207, EPI_ISL_2250211	Royal Darwin Hospital Pathology	Microbiological Diagnostic Unit Public Health Laboratory (MDU-PHL)	Meumann, E., Caly L., Seemann T., Sait, M.L., Druce J., Sherry, N.L.
EPI_ISL_2250230	Microbiological Diagnostic Unit - Public Health Laboratory (MDU-PHL)	Microbiological Diagnostic Unit Public Health Laboratory (MDU-PHL)	Seemann T., Sait, M.L., Sherry, N.L.
EPI_ISL_2253118	SARS-CoV-2 testing team, National Institute of Infectious Diseases	Pathogen Genomics Center, National Institute of Infectious Diseases	Tsuyoshi Sekizuka, Kentaro Itokawa, Rina Tanaka, Masanori Hashino, Nozomu Hanaoka, Masumichi Saito, Naomi Nojiri, Hazuka Y Furihata, Sana Uchikoba, Tsuguto Fujimoto, Makoto Kuroda
EPI_ISL_2259579, EPI_ISL_2259582	Eurofins LifeCodexx GmbH	Robert Koch Institute	unknown
EPI_ISL_2262288	RSPI Prof. Dr. Sulianti Saroso	National Institute of Health Research and Development	Hana Apsari Pawestri, Kartika Dewi Puspa, Arie Ardiansyah Nugraha, Subangkit, Hartanti Dian Ikawati, Triyani Soekarso, Krisna Pangesti, Nelly Puspandari, Vivi Setiawaty
EPI_ISL_2274032	Centro Nacional de Enfermidades Tropicales (CENETROP)	Laboratory of Respiratory Viruses and Measles, Oswaldo Cruz Institute, FIOCRUZ	Paola Resende, Roxana Loayza, Cinthia Avila, Luciana Appolinario, Fernando Motta, Anna Carolina Paixao, Ana Carolina Mendonca, Alice Sampaio Rocha, Taina Venas, Elisa Cavalcante Pereira, Renata Serrano Lopes, Marilda Siqueira on behalf of the Fiocruz COVID-19 Genomic Surveillance Network
EPI_ISL_2274244	PHV-FSS	PHV-FSS	Son Nguyen

EPI_ISL_2280634	Aegis Sciences Corporation	Centers for Disease Control and Prevention Division of Viral Diseases, Pathogen Discovery	Dakota Howard, Dhwani Batra, Peter W. Cook, Kara Moser, Adrian Paskey, Jason Caravas, Benjamin Rambo-Martin, Shatavia Morrison, Christopher Gulvick, Scott Sammons, Yvette Unoarumhi, Darlene Wagner, Matthew Schmerer, Cyndi Clark, Patrick Campbell, Rob Case, Vikramsinha Ghorpade, Holly Houdeshell, Ola Kvalvaag, Dillon Nall, Ethan Sanders, Alec Vest, Shaun Westlund, Matthew Hardison, Clinton R. Paden, Duncan MacCannell
EPI_ISL_2285858	National Influenza Centre	National Influenza Centre	William K. Ampofo, Michael Marks, Ivy A. Asante, Sharon Hsu, Benjamiin B. Lindsey, Benjamin H. Foulkes, Mildred Adusei-Poku, Linda Boatemaa, Lorreta Kwasah, Joseph Oliver-Commey, Ernest Asiedu, Franklin Asiedu-Bekoe, Gordon Awandare, Joyce Ngoi, Dennis Laryea, Mathew D. Parker, Thushan I de Silva,
EPI_ISL_2304093, EPI_ISL_2304094, EPI_ISL_2304112	Armed Forces Medical College Pune	National Centre For Cell Science - INSACOG	Dhiraj Paul, Mohak P Gujare, Shivang P. Bhanushali, Mitali Inamdar, Manoj Kumar Bhat, Ajay Pillai, INSACOG Consortium team, Yogesh Shouche
EPI_ISL_2313086	Department of Virology	Department of Virology	Massab Umair, Aamer Ikram, Muhammad Salman, Muhammad Ammar, Adnan Haider
EPI_ISL_2314284, EPI_ISL_2314288	Bioscientia MVZ Labor Karlsruhe GmbH	Robert Koch Institute	unknown
EPI_ISL_2321168	Royal Darwin Hospital Pathology	MDU-PHL	Meumann, E., Caly L., Seemann T., Sait, M.L., Druce J., Sherry, N.L.
EPI_ISL_2321171, EPI_ISL_2321175, EPI_ISL_2321177, EPI_ISL_2321180	Microbiological Diagnostic Unit - Public Health Laboratory (MDU-PHL)	MDU-PHL	Seemann T., Sait, M.L., Sherry, N.L.
EPI_ISL_2324948	National Institute of Public Health	National Institute of Public Health	Helena Jirincova, Jaromira Vecerova, Timotej Suri, Dusan Trnka, Alexander Nagy
EPI_ISL_2332727, EPI_ISL_2332758	National Centre For Cell Science	National Centre For Cell Science - INSACOG	Dhiraj Paul, Shivang P. Bhanushali, Mitali Inamdar, Mohak P Gujare, Manoj Kumar Bhat, Ajay Pillai, INSACOG Consortium team, Yogesh Shouche
EPI_ISL_2333520	Haugesund Hospital, laboratory for Medical Microbiology	Norwegian Institute of Public Health, Department of Virology	Kathrine Stene-Johansen, Kamilla Heddeland Instefjord, Hilde Elshaug, Garcia Llorente Ignacio, Jon Bråte, Engebretsen Serina Beate,Pedersen Benedikte Nevjen, Debech Nadia, Line Victoria Moen, Atiya R Ali,Marie Paulsen Madsen, Rasmus Riis Kopperud, Hilde Vollan, Karoline Bragstad, Olav Hungnes
EPI_ISL_2347406	Lighthouse Lab in Alderley Park	Wellcome Sanger Institute for the COVID-19 Genomics UK (COG-UK) Consortium	Jacquelyn Wynn, Mairead Hyland, The Lighthouse Lab in Alderley Park and Alex Alderton, Roberto Amato, Jeffrey Barrett, Sonia Goncalves, Ewan Harrison, David K. Jackson, Ian Johnston, Dominic Kwiatkowski, Cordelia Langford, John Sillitoe on behalf of the Wellcome Sanger Institute COVID-19 Surveillance Team
EPI_ISL_2348634	Rakai Health Sciences Program	MRC/UVRI & LSHTM Uganda Research Unit	Charles Ssuuna, Ronald Moses Galiwango, Steven J Reynolds, Dan Lule Bugembe, My V.T. Phan, Pontiano Kaleebu, Matthew Cotten
EPI_ISL_2349713, EPI_ISL_2349740, EPI_ISL_2349757, EPI_ISL_2349758, EPI_ISL_2349782	National Public Health Laboratory, National Centre for Infectious Diseases	National Public Health Laboratory, National Centre for Infectious Diseases	Tze Minn Mak, Zhenyang Zhou, Grace Jie Yin Ngan, Royce Ang, Lin Cui, Raymond Tzer Pin Lin
EPI_ISL_2349810, EPI_ISL_2349852	National Public Health Laboratory, National Centre for Infectious Diseases	National Public Health Laboratory, National Centre for Infectious Diseases	Tze Minn Mak, Zhenyang Zhou, Royce Ang, Lin Cui, Raymond Tzer Pin Lin
EPI_ISL_2360252	Shahid Ghodossi Laboratory	National Influenza Center	J Yavarian,K Sadeghi, NZ Shafiei Jandaghi, V Salimi, A Nejati, N Ghavvami,F Ajaminejad and T Mokhtari Azad
EPI_ISL_2360253	Maku Molecular Laboratory	National Influenza Center	A Nejati, J Yavarian,K Sadeghi, NZ Shafiei Jandaghi, V Salimi, N Ghavvami,F Ajaminejad and T Mokhtari Azad
EPI_ISL_2360254	Shahid Ghodossi Laboratory	National Influenza Center	K Sadeghi, A Nejati, J Yavarian, NZ Shafiei Jandaghi, V Salimi,F Ajaminejad,N Ghavvami and T Mokhtari Azad
EPI_ISL_2360256	Boshehr University of Medical Sciences	National Influenza Center	NZ Shafiei Jandaghi, V Salimi, A Nejati, K Sadeghi, J Yavarian, Mostafa Salehi-Vaziri, F Ajaminejad,N Ghavvami and T Mokhtari Azad
EPI_ISL_2360257	Boshehr University of Medical Sciences	National Influenza Center	V Salimi,NZ Shafiei Jandaghi, J Yavarian, A Nejati, K Sadeghi,Mostafa Salehi-Vaziri , N Ghavvami,F Ajaminejad and T Mokhtari Azad
EPI_ISL_2362624	Platform BIS UZA/UAntwerpen	Labo Klinische Biologie, UZA	Marie Le Mercier, Jasmine Coppens, Basil Britto Xavier, Christine Lammens, Veerle Matheeussen, Herman Goossens
EPI_ISL_2363824	SYNLAB Suomi	Expert Microbiology, National Institute for Health and Welfare	Soile Blomqvist, Jani Halkilahti, Kirsi Liitsola, Haider al-Hello, Päivi Laurila, Erika Lindh, Teemu Smura, Sari Hannula, Pekka Ellonen, Niina Ikonen, Carita Savolainen-Kopra
EPI_ISL_2363998	TYKS, Kliininen mikrobiologia	Expert Microbiology, National Institute for Health and Welfare	Soile Blomqvist, Jani Halkilahti, Kirsi Liitsola, Haider al-Hello, Päivi Laurila, Erika Lindh, Teemu Smura, Sari Hannula, Pekka Ellonen, Niina Ikonen, Carita Savolainen-Kopra
EPI_ISL_2367836	Quest Diagnostics Incorporated	Centers for Disease Control and Prevention Division of Viral Diseases, Pathogen Discovery	Dakota Howard, Dhwani Batra, Peter W. Cook, Kara Moser, Adrian Paskey, Jason Caravas, Benjamin Rambo-Martin, Shatavia Morrison, Christopher Gulvick, Scott Sammons, Yvette Unoarumhi, Darlene Wagner, Matthew Schmerer, S. H. Rosenthal, A. Gerasimova, R. M. Kagan, B. Anderson, M. Hua, Y. Liu, L.E. Bernstein, K.E. Livingston, A. Perez, I. A. Shlyakhter, R. V. Rolando, R. Owen, P. Tanpaiboon, F. Lacbawan, Clinton R. Paden, Duncan MacCannell
EPI_ISL_2370237, EPI_ISL_2370507	Aegis Sciences Corporation	Centers for Disease Control and Prevention Division of Viral Diseases, Pathogen Discovery	Dakota Howard, Dhwani Batra, Peter W. Cook, Kara Moser, Adrian Paskey, Jason Caravas, Benjamin Rambo-Martin, Shatavia Morrison, Christopher Gulvick, Scott Sammons, Yvette Unoarumhi, Darlene Wagner, Matthew Schmerer, Cyndi Clark, Patrick Campbell, Rob Case, Vikramsinha Ghorpade, Holly Houdeshell, Ola Kvalvaag, Dillon Nall, Ethan Sanders, Alec Vest, Shaun Westlund, Matthew Hardison, Clinton R. Paden, Duncan MacCannell
EPI_ISL_2372267	OUCRU	OUCRU	Nguyen Van Vinh Chau, Nguyen To Anh, Nguyen Thi thu Hong, Nghiem My Ngoc, Huynh Trung Trieu, Le Nguyen Truc Nhu, Nguyen Thi Han Ny, Lam Minh Yen, Ngo Ngoc Quang Minh, Nguyen Thanh Phong, Nguyen Thanh Truong, Le Thi Thu Huong, Tran Nguyen Hoang Tu, Le Manh Hung, Tran Tan Thanh, Nguyen Thanh Dung, Nguyen Tri Dung, Guy Thwaites, Le Van Tan
EPI_ISL_2379270, EPI_ISL_2379272, EPI_ISL_2379279, EPI_ISL_2379281, EPI_ISL_2379282, EPI_ISL_2379288, EPI_ISL_2379289, EPI_ISL_2379290, EPI_ISL_2379291	Microbiological Diagnostic Unit - Public Health Laboratory (MDU-PHL)	MDU-PHL	Seemann T., Sait, M.L., Sherry, N.L.
EPI_ISL_2379650	Institute for Medical Research, Infectious Disease Research Centre, National Institutes of Health, Ministry of Health Malaysia	Institute for Medical Research, Infectious Disease Research Centre, National Institutes of Health, Ministry of Health Malaysia	Suppiah J, Kamel K, Mohd Zawawi Z, Azizan MA, Ramly N, Robert F, Thayan R
EPI_ISL_2379748	National Heart Institute	Institute for Medical Research, Infectious Disease Research Centre, National Institutes of Health, Ministry of Health Malaysia	Suppiah J, Kamel K, Mohd Zawawi Z, Azizan MA, Ramly N, Robert F, Thayan R
EPI_ISL_2382973	National Centre For Cell Science	National Centre For Cell Science-INSACOG	Dhiraj Paul, Shivang P. Bhanushali, Mohak P Gujare, Mitali Inamdar, Manoj Kumar Bhat, Ajay Pillai, INSACOG Consortium team, Yogesh Shouche
EPI_ISL_410301	National Influenza Centre, National Public Health Laboratory, Kathmandu, Nepal	The University of Hong Kong	Ranjit Sah , Runa Jha, Daniel Chu, Haogao Gu, Malik Peiris, Anup Bastola, Alfonso J. Rodriguez-Morales, Bibek Kumar Lal, Basu Dev Pandey, Leo Poon
EPI_ISL_454409	UPMC Clinical Microbiology Laboratory	Microbial Genome Sequencing Center, Microbial Genomic Epidemiological Laboratory	Mustapha M. Mustapha, Jane W. Marsh, Dan Snyder, Marissa P. Griffith, Stephanie L. Mitchell, Vatsala R. Srinivasa, Kady D. Waggle, Chinelo Ezeonwuku, Vaughn S. Cooper, Lee H. Harrison
EPI_ISL_454974	Wuhan Chain Medical Labs (CMLabs)	State Key Laboratory of Biotherapy of Sichuan University	Baowen Du, Minjin Wang, Chao Tang, Chuan Chen, Yongzhao Zhou, Mingxia Yu, Hancheng Wei, Weimin Li, Jing-wen Lin, Jia Geng, Binwu Ying, Lu Chen
EPI_ISL_486421	Centrl laboratorija	Latvian Biomedical Research and Study Centre	Ivars Silamielis, Kaspars Megnis, Monta Ustinova, ikita Zrelovs, Vita Rovte, Stella Lapia, Jana Oste, Marta Priedte, Uga Dumpis, Jnis Kloviš
EPI_ISL_636987	Virology Lab, National Institute for Biomedical Research (INRB)	Project group Epidemology of Highly Pathogenic Microorganisms, Robert Koch-Institute	Jean-Jacques Muyembe Tamfum, Steve Ahuka-Mundeke, Eddy Kinganda-Lusamaki, Gabriel Mbunsu, Sheila Makiala, Essia Belarbi, Jasmin Schlotterbeck, Grit Schubert, Fabian Leendertz
EPI_ISL_710575	Hôpital Fattouma-Bourguiba de Monastir	Laboratoire des Procédés de Criblage Moléculaire et Cellulaire-Centre de Biotechnologie de Sfax	Souissi,A., Abid,N., Ben Ayed,I., Gargouri,S., Abdelmoulah,F.,Elargoubi,A., Smeti,I., Bensaid,M., Stambouli,N., Kharat,N., Ajili,F., Fki-berrajah,L., Mhalla,S., Chtourou,A., Gaaloul,I., Nabli,A., Turki,M., Aouni,M., Hammami,A., Mastouri,M., Karray Hakim,H., Kamoun,S., Rebai,A. and Masmoudi,S.
EPI_ISL_729867	Laboratorio de Referencia Nacional de Virus Respiratorios, Instituto Nacional de Salud Peru	Laboratorio de Genómica Microbiana, Universidad Peruana Cayetano Heredia	Pablo Tsukayama, Alejandra Dávila-Barclay, Luis González, Guillermo Salvatierra, Pedro E. Romero, Brenda Ayzanoa, Janet Huancachoque, Pool Marcos, Marco Galarza, Priscila Lope, Nancy Rojas

EPI_ISL_740469	Laboratoire national de santé, Microbiology, Virology	Laboratoire national de santé, Microbiology, Microbial Genomics Platform	Anke Wienecke-Baldacchino, Catherine Ragimbeau, Tamir Abdelrahman, Jessica Tapp, Fatu Djabi
EPI_ISL_859733	BTC, Khalifa University	BTC, Khalifa University	Al Safar et al
EPI_ISL_887445	Instituto Nacional de Saude (INS), Mozambique	KRISP, KZN Research Innovation and Sequencing Platform	Nalia Ismael, Nadia Sitoe, Paulo Arnaldo, Nedio Mabunda, Giandhari J, Pillay S, Tegally H, Wilkinson E, de Oliveira T
EPI_ISL_895845	Molecular biology division, Institute of Clinical Biochemistry and Diagnostics, Charles University, Faculty of Medicine in Hradec Králové and University Hospital Hradec Králové	Molecular biology division, Institute of Clinical Biochemistry and Diagnostics, Charles University, Faculty of Medicine in Hradec Králové and University Hospital Hradec Králové	Helena Kovaíková, Petr Brož, Ivana Baranová, Kateina Hrochová, Tereza Baková, Jitka Novotná, Kateina Pehlíková, Vladimír Palika. Cooperation project with BioVendor-R&D and bioinformatics company BIOXSYS s r.o.
